# A hybrid model integrating long short-term memory with adaptive genetic algorithm based on individual ranking for stock index prediction

**DOI:** 10.1371/journal.pone.0272637

**Published:** 2022-08-17

**Authors:** Xiaohua Zeng, Jieping Cai, Changzhou Liang, Chiping Yuan

**Affiliations:** 1 School of Economics and Trade, Guangzhou Xinhua University, Dongguan, China; 2 Lingnan College, Sun Yat-Sen University, Guangzhou, China; Shandong Normal University, CHINA

## Abstract

Modeling and forecasting stock prices have been important financial research topics in academia. This study seeks to determine whether improvements can be achieved by forecasting a stock index using a hybrid model and incorporating financial variables. We extend the literature on stock market forecasting by applying a hybrid model that combines wavelet transform (WT), long short-term memory (LSTM), and an adaptive genetic algorithm (AGA) based on individual ranking to predict stock indices for the Dow Jones Industrial Average (DJIA) index of the New York Stock Exchange, Standard & Poor’s 500 (S&P 500) index, Nikkei 225 index of Tokyo, Hang Seng Index of Hong Kong market, CSI300 index of Chinese mainland stock market, and NIFTY50 index of India. The results indicate an overall improvement in forecasting of the stock index using the AGA-LSTM model compared to the benchmark models. The evaluation indicators prove that this model has a higher prediction accuracy when forecasting six stock indices.

## 1. Introduction

Stock market forecasting is one of the most challenging research topics in the financial field. The time series of the stock market has typical characteristics of nonlinearity, high noise, and dynamic change. A company’s fundamental information, news, investors’ psychological state, industry background, macro policies, and other factors may affect the violent fluctuation of the stock. This high volatility makes it difficult to predict stock markets. The advantage of traditional econometric models is the processing of linear data based on strict basic assumptions. The models include the autoregressive integrated moving average (ARIMA) and generalized autoregressive conditional heteroskedasticity (GARCH) models, which have proven to be effective under these premises [1]. However, the linear model cannot accurately reflect the real distribution of stock data or solve complex financial data series problems. Therefore, traditional econometric methods cannot achieve the best prediction when dealing with nonlinear, non-parametric, and massive data [2]. In recent years, with the development of artificial intelligence technology, an increasing number of researchers have applied machine learning models to stock market predictions. Machine learning methods can better deal with nonlinear data, which is conducive to improving the accuracy of stock prediction models [3]. Common machine learning methods include deep learning networks (DLNs), BP neural networks, genetic algorithms (GA), support vector machines (SVM), and random forests (RF) [4, 5]. Recurrent neural networks (RNN), convolutional neural networks (CNN), and feedforward neural networks (FFNN) are the three major categories of DLNs [6, 7].

Ersan, Nishioka, and Scherp compared the K-nearest neighbor (KNN), ANN, and SVM in financial time series forecasting. The results indicated that the prediction effects of the KNN and ANN models were better than that of the SVM model [8]. However, the drawback of ANNs is that the temporal influence of historical trading data is not considered or retained when the model is applied to forecasting financial time series [9]. Other DLN types, especially the recurrent neural networks (RNNs), have been widely proposed by expert researchers [10, 11]. Unlike ANNs, past information is considered in the feedback connection available in the RNNs’ architecture. Recent studies that compare the predictive performance of RNN models with those of ANN models indicate that RNN models predominate over ANN models [12, 13]. Long short-term memory (LSTM) is one of the most successful variants of RNN and has been proven to be an efficient model for forecasting financial time series [14]. Compared with RNN, LSTM improves the gate structure, which remembers past information of longer time series with no gradient disappearance or explosion. LSTM can selectively learn useful hidden information from historical data to enjoy a high level of adaptability to sequential data [15]. Moghar and Hamiche utilized LSTM for stock price prediction and achieved satisfactory accuracy [16]. Fischer and Krauss used LSTM to predict the yield of S&P 500 stock. The findings showed that LSTM was better than RF, DNN, and logistic regression (LOG) in predictive performance [17]. LSTM is recognized as an excellent model for time-series analysis. However, LSTM’s architecture cannot extract time–frequency information, which is a limitation of modeling the frequency domain of the time series.

Wavelet transform (WT) effectively obviates this problem using the principle of localized short-time Fourier transform (FT). The original time series can be decomposed into different frequency segmentations using the WT. The frequency bands did not overlap with each other. The decomposed frequency range includes all frequency bands of the original time series. In recent studies, some researchers have combined WT into LSTM to refine the model by extracting multi-frequency features of time series to forecast the stock index [12, 18, 19].

Hyperparameters of neural network models, such as the input window length, number and size of hidden layers, number of neurons, learning rate, dropout rate, etc., are important factors affecting the accuracy and convergence of stock prediction models. Many optimization-based approaches have been employed to tune the hyperparameters of neural network models, such as the Bayesian optimization algorithm [20], grid search (GS), and random search (RS) [21]. GS is applicable when there are few tuning parameters within a small search range, whereas RS blindly searches for hyperparameters [22]. To solve the problems of RS’s blind search and GS’s over-search, various meta-heuristic algorithms such as GA and particle swarm optimization (PSO) are preferentially applied to optimize hyper parameters. Junior and Yen utilized the PSO algorithm, which uses only 30 particles and 20 iterations to find hyper-parameters, demonstrating the superiority of the PSO-CNN model [23]. Chang et al. applied a GA to tune the connection weight of a partially connected neural network to predict stock trends [24]. Chung and Shin proposed a GA to tune the time window size and number of units of the LSTM network to improve the performance of the stock market forecasting model, and the experimental results proved that the GA is an effective method for finding optimal solutions with large search problems [25, 26]. Jaddi et al. integrated GA with ANN to explore hyperparameters such as the number of hidden layers, different numbers of nodes for each layer, weights, and biases [27].

GA is a parallel and global search algorithm that can effectively solve complex problems by simulating the genetic operators to imitate the biological process of reproduction. Crossover and mutation are the most important phases in GA operation. The crossover process in the GA may lead to local minima, whereas a mutation is proposed to overcome this issue because it randomly generates a new chromosome called a mutant [28]. GA uses a fixed crossover rate and mutation rate during the evolutionary process. Individuals with inferior fitness can be quickly screened during the early stages of evolution. However, this method suffers from a premature convergence. Ho proposed a sequential optimization method to quantitatively evaluate an optimization method based on random probability [29]. The adaptive genetic algorithm (AGA) based on individual ranking can increase the crossover rate and mutation rate in the middle and late stages of population evolution and accelerate the convergence speed of the population to the optimal solution.

Fixed crossover rate and mutation rate are adopted in canonical genetic algorithms. The advantage of fixed crossover rate and mutation rate that is that the individuals with poor fitness can be quickly screened out in the early stage of evolution. These algorithms cost little computation resources and gain fast convergence speed. However, there is the problem of premature convergence. AGA is a sequential optimization method to quantitatively evaluate the optimization method from the perspective of probability. This random optimization algorithm can be suitable for solving the problem. By using AGA, the average fitness value and all fitness values in the population are calculated and combined into a new fitness value matrix after initialization, selection and other operations. Then, the fitness values, that is, the ranking number of the maximum, minimum and average fitness values, of all individuals are ranked in the new matrix. The ranking number is substituted instead of the specific fixed fitness value into the adaptive calculation formula of crossover rate and mutation rate. By this method, the crossover rate and mutation rate are determined by the ranking position of individual fitness value in the population instead of the actual fitness value. Therefore, the sorting number is used to update the crossover and mutation probability adaptively. The adaptive strategy can increase the crossover rate and mutation rate in the middle and late stage of population evolution, improve the optimization ability of the algorithm, help the algorithm jump out of the local optimal solution and accelerate the convergence speed of the population to the optimal solution.

A hybrid model integrating long short-term memory and adaptive genetic algorithm based on individual ranking (AGA) is proposed in this study. AGA is adopted to optimize the network structure of the LSTM model, the number of neurons in the LSTM layer, the number of neurons in the full connection layer, the exit rate and the training cycle of the neural network. AGA-LSTM model is applied to predict stock price. The main contributions of this study are as follows: (1) High accuracy. AGA-LSTM model is proposed to train the hyperparametric combination to find the optimal hyperparametric combination. Compared with other models, AGA-LSTM model achieves higher accuracy in predicting stock prices. (2) High computational efficiency. The optimization methods of real number coding and individual sequencing are applied in AGA. These optimization strategies help the crossover and mutation probability of genes adjust adaptively. As the difference of each individual’s fitness is small, the adaptive algorithm based on individual fitness ranking can improve the speed of finding the optimal solution effectively. AGA-LSTM model has high computational efficiency. (3) Better convergence. The elitist preservation is adopted to ensures the optimal individuals that emerge during the evolutionary process will not lost or destroyed in the processes of selection, crossover and mutation operations. The strategy enhances the ability of the algorithm to converge to the global optimum. The experimental results show that the algorithm has better convergence to the global optimization.

This study will optimize the LSTM model by employing the adaptive genetic algorithm (AGA) based on individual ranking to obtain the superior prediction effect. The model proposed in this study consists of three parts: wavelet transform (WT), adaptive genetic algorithm based on individual sorting (AGA), and long and short-term memory (LSTM). WT was applied for the denoising time series. AGA optimizes the network structure, number of neurons, training times, and forgetting rate of the LSTM model through real number coding, crossover operators, and mutation operators based on individual ranking. The prediction accuracy was evaluated using five measurements: mean square error (MSE) [30–32], root mean square error (RMSE) [31–33], mean absolute error (MAE) [30–33], mean absolute percentage error (MAPE) [30–32], and goodness of fit (R^2^) of the model [33, 34].

According to the efficient market hypothesis (EMH), the degree of market development is heterogeneous. Six stock indices were used to test the prediction accuracy of the model. These indices include the Dow Jones Industrial Average (DJIA) index of the New York Stock Exchange, Standard & Poor’s 500 (S&P 500) index, Nikkei 225 index of Tokyo, Hang Seng Index of Hong Kong market, CSI300 index of Chinese Mainland stock market, and Nifty50 index of India. These six stock indices represent three different developmental stages of the stock market. For example, the New York Stock Market is the most mature and largest exchange in the world. It is considered the most developed and efficient market. The stock markets in Hong Kong and Tokyo are in the middle stage between efficient and inefficient markets. In contrast, the stock markets in mainland China and India are generally considered developing markets; therefore, we can evaluate the effectiveness of our model by testing the stock market in different developmental stages.

The remainder of this paper is organized as follows. Section 2 introduces the methodology applied in this study, including the LSTM, GA, and AGA. Section 3 presents the hybrid model and introduces the details of the experimental design. Section 4 summarizes the experimental results and compares them to those of the benchmark model. Finally, Section 5 summarizes the research.

## 2. Methodology

### 2.1 LSTM

**Fig 1:**
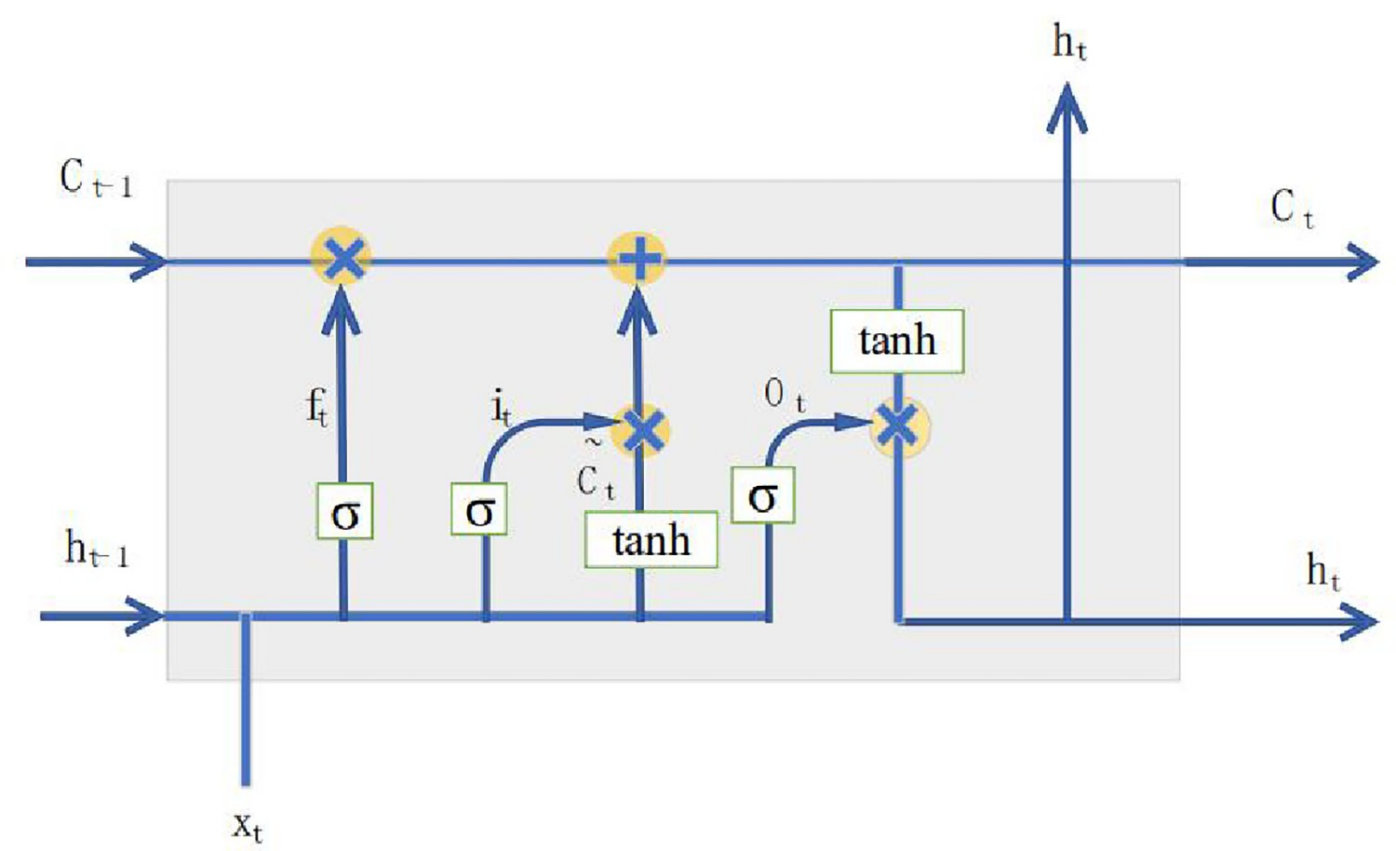
The repeating module in an LSTM.

The RNN structures enable the learning of temporal patterns for sequential data. However, RNNs cannot memorize temporal patterns in previous deep feedforward networks over a long period of time because of the gradient disappearance or gradient explosion of RNNs [35]. The LSTM unit was proposed as an RNN variant of RNNs learning sequential patterns [36]. LSTMs have gating mechanisms that can selectively learn and retain temporal patterns over a longer time span. Fig 1 represents the repeating module in an LSTM consisting of a memory cell and gate.

The gating mechanisms, consisting of a sigmoid activation function and point multiplication operation, regulate the flow of information. The three gates are called the input, forget, and output gates, respectively, and the calculations for each gate are performed using the following formulas.

The input gate i_t_ shown in Formula 1 and the candidate value of the memory cells ${\tilde{C}}_{t}$ shown in Formula 2 update the selected information. The calculation is performed by multiplying the two vectors created by the input gate layer and the *tanh* layer.


$$i_{t}=\sigma (W_{i}∙[h_{t-1},x_{t}]+b_{i}),$$ (1)

$${\tilde{C}}_{t}=tanh(W_{C}∙[h_{t-1},x_{t}]+b_{C}).$$ (2)

The forget gate can be written as

$$f_{t}=\sigma (W_{f}*[h_{t-1},x_{t}]+b_{f}),$$ (3)

where σ is the sigmoid activation function, W_f_ represents the connection weight of the previous output, b_f_ is the bias vector, h_t−1_ and x_t_ correspond to the previous output and the current input, respectively.

The output gate updates the temporal state by adding information from the input and forget gates [37]. *o*_*t*_ and *C*_*t*_ are the values of the output gate and memory cell at time t, respectively.

The new updated state of the memory cell is shown as:

$$C_{t}=f_{t}*C_{t-1}+i_{t}*{\tilde{C}}_{t}$$ (4)

The value of the output gate was calculated based on the new state of the memory cell.


$$o_{t}=\sigma (W_{o}[h_{t-1},x_{t}]+b_{0}).$$ (5)

The final output value of cell is calculated by

$$h_{t}=o_{t}*tanh(C_{t}).$$ (6)

**Fig 2:**
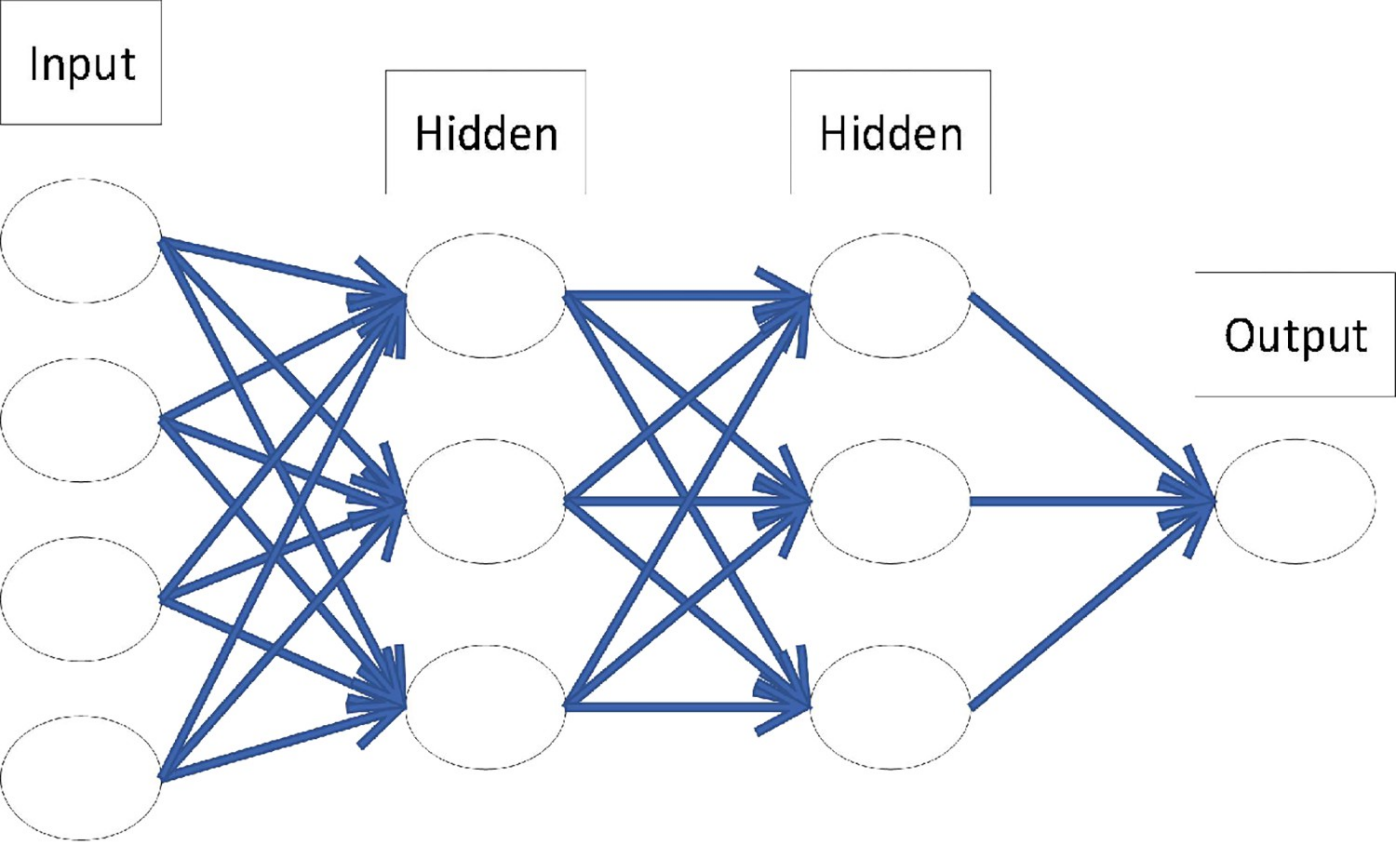
The LSTM network structure.

The output value of the memory cell is determined by four units: input gate, output gate, forget gate, and self-recurrent neuron. The input gate determines whether the input signal can alter the state of the memory cell. The output gate controls the updated state of the memory cell. Furthermore, the forget gate decides to retain or discard its previous state, and the LSTM architecture is an effective solution for combating vanishing gradients confronted with RNNs using memory cells. Fig 2 shows the LSTM network structure connected to each LSTM unit with a chain structure.

The training process of the LSTM model includes three steps. First, the output value of the LSTM unit was calculated using the forward propagation method, and the error value was back-propagated. Second, the weight gradient is calculated based on the error value. Subsequently, the optimization algorithm is applied for gradient descent, and the weight is continuously updated. Owing to this repeating process, the LSTM architecture is capable of learning long-term patterns at low computational costs [38].

Stochastic gradient descent (SGD), adaptive gradient algorithm (AdaGrad), and adaptive moment estimation algorithm (Adam) are three typical optimization algorithms used in neural networks. The SGD maintains a single learning rate. Conversely, Adam can obtain an independent adaptive learning rate for each parameter based on the calculations of both the first-order moment estimation and second-order moment estimation [39]. However, long training times and overfitting problems occurred as the number of network layers increased. Adding a dropout rate and omitting a certain ratio of feature detectors randomly in each training case can effectively reduce overfitting [40].

### 2.2 GA

The GA is a metaheuristic and stochastic optimization method that mimics the evolution mechanism of natural selection according to “the survival of the fittest” principle [41]. The GA simulates the natural evolution process through chromosomes, populations, offspring, and parents to search for the optimal solution. The basic steps in heredity include gene coding, generation of the primary population, chromosome selection, chromosome crossover, and chromosome mutation. The GA continuously produces new offspring to determine the optimal solution by gradually transforming the chromosomes that are the candidate solutions to the given problem.

The definition of the fitness function, crossover rate, and mutation rate are crucial factors in the GA implementation process. The fitness function can be considered as the degree of adaptation of individuals in the population in nature. The higher the fitness, the greater the chance of reproduction, and vice versa. Therefore, determination of the fitness function is directly related to the search for an optimal solution [28]. The crossover and mutation rates directly affect the optimization ability of the genetic algorithm. The crossover operation is at the core of GA. The large crossover rate causes the pattern of old individuals to be easily destroyed to produce new individuals faster. The recombination of excellent individual genes produces superior individuals with a certain probability. Mutation is a key factor that helps the algorithm jump out of local optimization. However, a low variation rate cannot produce a new pattern structure. However, the high mutation rate makes the algorithm a random-search algorithm.

### 2.3 AGA

The GA uses a fixed crossover rate and mutation rate, which can quickly screen out individuals with poor fitness in the early stages of evolution. However, it is easy to destroy excellent individuals in the late stage of evolution, which leads to premature convergence. The mechanism of adaptive genetic algorithm (AGA) proposes that the crossover rate and mutation rate should change based on the individual fitness value to improve the optimization ability and accelerate the convergence speed.

Srinivas and Patnaik proposed that the crossover rate and mutation rate should be dynamically adjusted according to individual adaptability to balance the search and randomness [42]. The concentration of population fitness and rare diversity restrict most individuals in the population within the local optimal solution set. More concentrated individuals in the population can evolve further by increasing the crossover and mutation rates, which enhances the optimization ability of the algorithm. The dispersion and diversity of population fitness cause the population to scatter in the solution space. The convergence of the individual fitness value towards the optimal solution set can be promoted by reducing the crossover and mutation rates, which accelerates the convergence of the algorithm. However, this adaptive method stagnated during the early stages of the evolution. In addition, when the crossover and mutation rates are zero, superior individuals cannot undergo benign evolution, which makes the evolution process easy to fall into local optimization. The crossover and mutation rates of AGA were calculated as follows.: *F*_max_ is the maximum fitness value and *F*_avg_ is the average fitness value of the population.


$$P_{c}=\{\begin{array}{c} \frac{K_{1}\times (F_{\max}-F^{\prime })}{F_{\max}-F_{avg}},F^{\prime }\geq F_{avg}\\ K_{2},F^{\prime }<F_{avg} \end{array}$$ (7)

$$P_{m}=\{\begin{array}{c} \frac{K_{3}\times (F_{\max}-F^{\prime })}{F_{\max}-F_{avg}},F^{\prime }\geq F_{avg}\\ K_{4},F<F_{avg} \end{array}$$ (8)

To solve local optimization problems, Zhang et al. proposed improved calculation formulas for AGA [43]. The improved formulas improve the crossover and mutation rates of superior individuals, which avoids the crossover and mutation rate of individuals with the maximum fitness value from zero. This prevents the potential optimal solution in the initial stage of population evolution from stopping evolution, which makes the algorithm jump out of the local optimal solution to obtain the global optimal solution. The improved formulas are as follows.


$$P_{e}=\{\begin{array}{c} P_{e1}\times \frac{1}{(P_{e1}-P_{e2})+e^{\frac{F^{\prime }-F_{avg}}{F_{\max}-F_{avg}}}},F^{\prime }\geq F_{avg}\\ K_{1}\times P_{e1},F^{\prime }<F_{avg} \end{array}$$ (9)

$$P_{m}=\{\begin{array}{c} P_{m1}\times \frac{1}{(P_{m1}-P_{m2})+e^{\frac{F^{\prime }-F_{avg}}{F_{\max}-F_{avg}}}},F^{\prime }\geq F_{avg}\\ K_{2}\times P_{m1},F<F_{avg} \end{array}$$ (10)

Qu et al. suggested that the crossover and mutation rates of superior individuals should be reduced as much as possible to preserve them. Meanwhile, the cross-mutation rate should be increased to change the inferior state as much as possible in inferior individuals [44]. The crossover and mutation rates should be adaptive to different iteration periods. A larger crossover rate and mutation rate can expand the range of solutions and quickly find the optimal solution set in the early stages of population iteration. The smaller crossover and mutation rates cause the population to converge quickly in the optimal solution set in the later stages of the iteration.

Ho proposed an optimization method based on sorting numbers, which uses ordinal numbers instead of cardinal values for optimization [29]. The crossover and mutation rates obtained by this method depend on the ordinal number of individual fitness values in the population, rather than the actual fitness value. The algorithm combines the average fitness value and all the fitness values in the population into a matrix. The ranking number of the maximum, minimum, and average fitness values in the matrix can then be obtained by ranking the fitness values of all individuals. Finally, the ranking number is substituted into the calculation formula of the crossover rate and mutation rate, instead of the specific fitness value. The formulas for the adaptive crossover and mutation rates are as follows:

$$P_{m}=\{\begin{array}{c} P_{m1}\times \frac{1}{(P_{m1}-P_{m2})+e^{\frac{N1-N2}{N3-N2}}},N_{1}\geq N_{2}\\ 0.9,N_{1}<N_{2} \end{array}$$ (11)

$$P_{e}=\{\begin{array}{c} P_{e1}\times \frac{1}{(P_{e1}-P_{e2})+e^{\frac{N1-N2}{N3-N2}}},N_{1}\geq N_{2}\\ 0.9,N_{1}<N_{2} \end{array}$$ (12)

*N*_1_ is the ranking number of larger parent fitness values. *N*_2_ is the ranking number of the average fitness value, and *N*_3_ is the ranking number of the maximum fitness value. The ranking method appropriately increases the crossover and mutation rates in the later stages of the population. *P*_c1_ is equal to 0.9 or 1. *P*_c2_ takes a value in the interval [0.5,1] to adjust the crossover rate. The *P*_*m*1_ is equal to 0.09 or 0.1. *P*_c2_ takes a value in the interval [0.05,0.1] to adjust the crossover rate.

### 2.4 A hybrid prediction model based on LSTM approach and AGA

**Fig 3:**
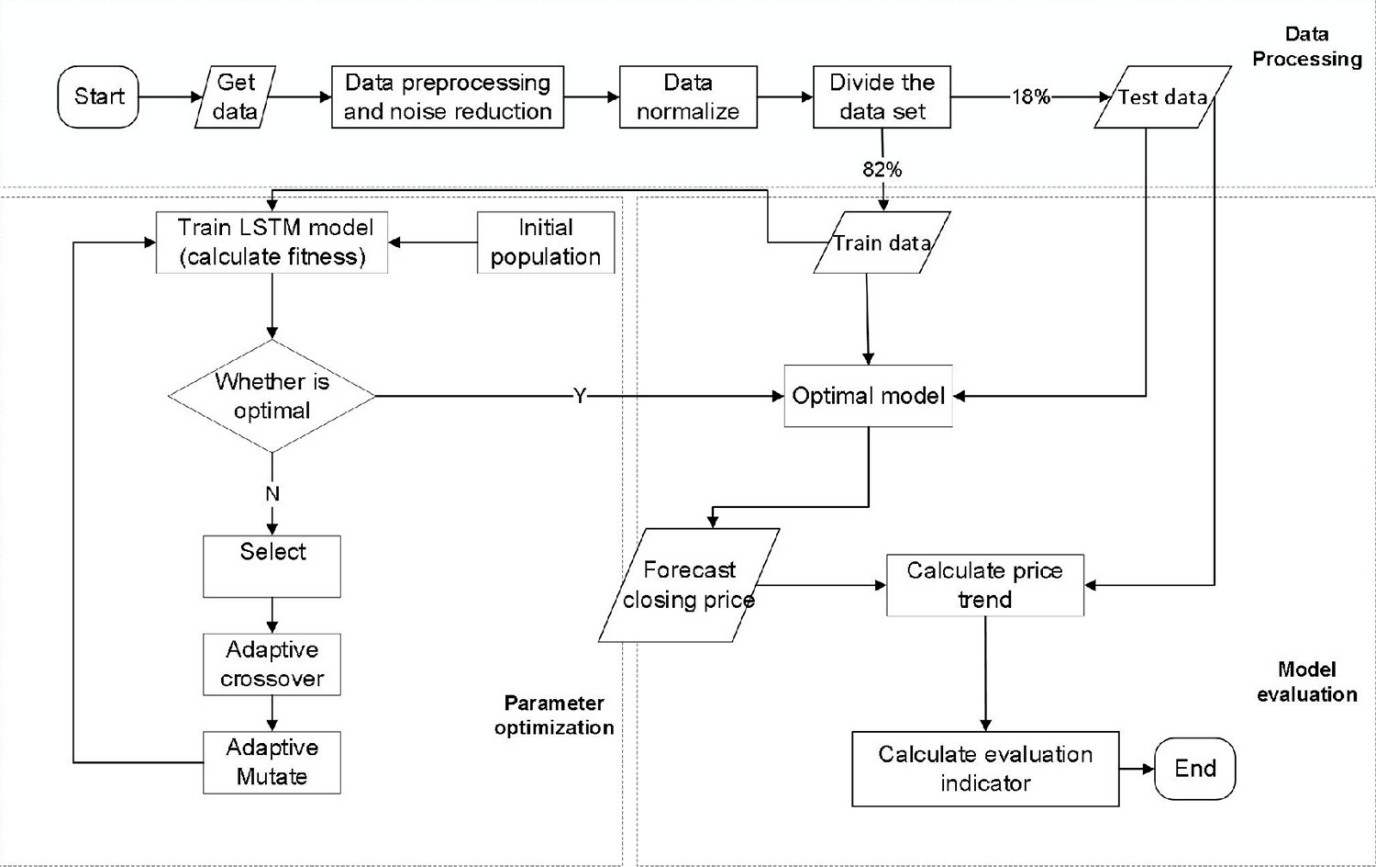
Flowchart of AGA-LSTM model.

According to recent related studies, LSTM is considered suitable for stock market predictions. In this study, we propose a hybrid model that integrates LSTM and AGA for stock index prediction. This model comprises three main components: data processing, parameter optimization, and model evaluation, as shown in Fig 3. The first component processes the historical stock-trading data used in the model. The second component employs AGA to optimize the parameters based on the LSTM neural network. Finally, the predictive accuracy of the hybrid model was evaluated by combining the optimization results. Furthermore, the model was compared with different benchmark models.

## 3. Research data and experiment

### 3.1 Dataset preparation and prepossessing

#### 3.1.1 Sample selection and predictive inputs

DJIA, S&P500, Hang Seng, Nikkei 225, CSI 300, and Nifty50 are the six stock indices chosen as samples, which represent the categories of markets; all six stock index datasets are from the WIND database (http://www.wind.com.cn) provided by Shanghai Wind Information Co., Ltd, CSMAR database (http://www.gtarsc.com) provided by Shenzhen GTA Education Tech. Ltd., and the global financial portal Investing.com. The time series for the daily datasets were from 2008/07/02 to 2016/09/30. We selected three sets of variables as the inputs. The first set of predictive inputs is the historical trading data of each index, including open, high, low, and close prices (OHLC variables), as well as the trading volume [17, 45, 46]. These raw prices record fundamental trading information for each index. The second set of predictive inputs consists of 12 technical indicators for each index [47, 48]. The details are as follows: moving average convergence divergence (MACD), commodity channel index (CCI), average true range (ATR), Bollinger band (BOLL), 20 day Exponential Moving average (EMA20), 5/10 day Moving average (MA5/MA10), 6/12 month momentum (MTM6/MTM12), price rate of change (ROC), stochastic momentum index (SMI), and Williams’s variable (WVAD). Technical indicators can summarize the behavior or trends in a time series. This representation is more appropriate than raw prices, which simplifies the machine learning models. The final set of predictive inputs is the macroeconomic indicators, including the exchange rate and interest rate, which are the most popular economic indicators used as variables [49, 50].

Interest rates have proven useful as predictive inputs [49]. Fundamental analysis uses economic indicators to understand how stock market changes are related to macroeconomic conditions, particularly the influence of the monetary market. As the US dollar plays the most important role in the international monetary market, the US dollar index is used as the proxy for the exchange rate in this study. Regarding the interest rate, the interbank offered rate in each market is appropriate as a proxy [49]. The federal funds rate in the US, Tokyo Interbank Offered Rate (TIBOR), Hong Kong Interbank Offered Rate (HIBOR), Shanghai Interbank Offered Rate (SHIBOR), and Mumbai Interbank Offered Rate (MIBOR) are used as predictive inputs. Tables 1–3 are the data examples.

**Table 1:** Summary statistics of selected input variables (historical trading data) (S&P500).

	Open	High	Low	Close	Volume
**count**	2080.00	2080.00	2080.00	2080.00	2080.00
**mean**	1509.99	1519.08	1500.19	1510.40	413532.54
**std**	414.25	413.72	414.86	414.37	110173.06
**min**	691.15	704.93	675.88	688.08	97120.50
**25%**	1175.41	1186.56	1163.73	1173.99	343623.73
**50%**	1402.42	1409.61	1397.01	1405.15	384082.60
**75%**	1942.26	1953.01	1930.53	1942.83	462497.69
**max**	2185.82	2189.56	2180.53	2185.70	1064193.53

**Table 2:** Summary statistics of selected input variables (technical indicators) (S&P500).

	CCI	ATR	BOLL	EMA20	MA10	MTM6	MA5	MTM12	ROC	SMI	WVAD
**count**	2080	2080	2080	2080	2080	2080	2080	2080	2080	2080	2080
**mean**	19.98	19.62	1505.45	1506.63	1508.55	2.29	1509.56	4.82	0.38	0.00	9.20E+7
**std**	94.31	10.37	410.58	411.08	413.07	33.57	413.84	47.08	3.78	0.03	2.28e+8
**min**	-270.12	6.20	750.69	743.38	708.31	-266.25	694.38	-273.75	-23.98	-0.22	-7.04E+8
**25%**	-58.28	13.28	1177.16	1177.41	1177.04	-12.42	1175.48	-18.79	-1.11	-0.01	-4.85E+7
**50%**	44.14	16.87	1392.74	1394.55	1397.54	6.03	1403.17	12.61	0.79	0.01	9.62E+7
**75%**	99.35	22.43	1948.35	1947.31	1943.03	21.96	1943.08	32.64	2.41	0.02	2.52E+8
**max**	201.21	93.46	2178.61	2176.41	2183.19	115.65	2185.25	128.03	18.33	0.13	7.08E+8

**Table 3:** Summary statistics of selected input variables (macroeconomic indicators) (S&P500).

	US DOLLAR INDEX	FEDERAL FUND
**count**	2080	2080
**mean**	83.7585	0.2213
**std**	7.1560	0.3424
**min**	72.1784	0.0574
**25%**	79.3798	0.0946
**50%**	81.0572	0.1365
**75%**	86.8313	0.1856
**max**	99.9594	2.9542

#### 3.1.2 Data denoising

**Fig 4:**
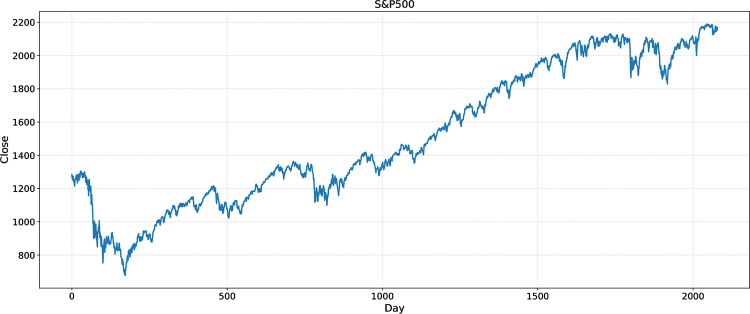
Time series for daily index of S&P500 for 2008/07/02-2016/09/30 before WT.

**Fig 5:**
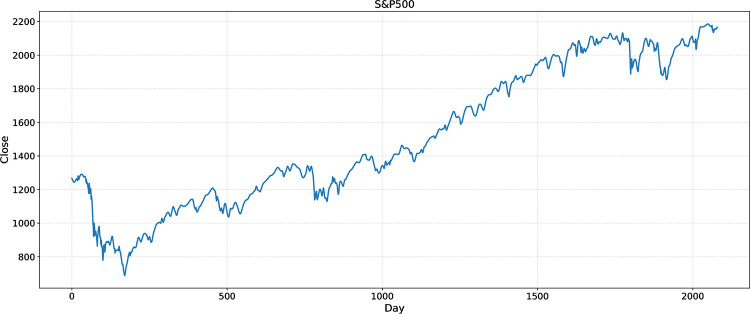
Time series for daily index of S&P500 for 2008/07/02-2016/09/30 after WT.

In this study, the Pywt Library in Python was used for wavelet transform to remove data noise from the six-stock index. Figs 4 and 5 are the closing price curves of the S&P500 before and after wavelet transform, respectively.

#### 3.1.3 Data normalization

Historical stock price data contain continuous variables with different measurement units for volume and price. Furthermore, some technical indicators include rate measurements. Data normalization is a crucial step that can utilize (13) to process different scaled features. Specifically, the normalization of input feature values is helpful for speeding up gradient descent convergence [26]. As data normalization preserves all relationships in the data precisely, it avoids bias [51].

$$x_{norm}=\frac{x-x_{min}}{x_{max}-x_{min}},$$ (13)

where, *x*_*norm*_ is the converted value. *x*_*max*_ is the maximum value of the sample and *x*_*min*_ is the minimum value of the sample.

Through data normalization, the original data sequence *x* = (*x*_1_, *x*_2_,…,*x*_*n*_}, was converted into a new data sequence *D* = (*d*_1_, *d*_2_,…,*d*_*n*_}. The normalized data were divided into training and test sets, *d*_*tr*_ = (*d*_1_, *d*_2_,…,*d*_*m*_} and *d*_*te*_ = (*d*_*m*+1_, *d*_*m*+2_,…,*d*_*n*_}, respectively. S is the timestamp of the data, which was set to 50. Therefore, the input data are as follows:

$$X=\{X_{1},X_{2},...,X_{s}\},$$ (14)

$$X_{i}=\{d_{i},d_{i+1},\ldots ,d_{m-S+p-1}\}\;s.t\;1\leq i\leq S;i,S\in N.$$ (15)

The actual and predicted closing prices are shown as follows.


$$Y=\{Y_{m+1},Y_{m+2},\ldots ,Y_{n}\},$$ (16)

$$y=\{y_{m+1},y_{m+2},\ldots ,y_{n}\}.$$ (17)

### 3.2 AGA-LSMT model training

#### 3.2.1 Real number coding

The AGA coding method used in this study was real number coding. This coding method has the function of fine-tuning and directly performs genetic operations on the performance of the solution, which can mine heuristic information related to the optimal solution of the problem.

**Fig 6:**
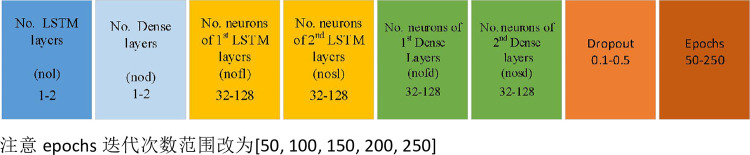
Chromosome of adaptive genetic algorithm.

Eight genes were identified for each individual. The first gene, with a step size of one, represents the number of LSTM layers. The number of layers ranged from one to two. The second gene, with a step size of 1, represents the number of fully connected layers, and the number of layers ranges from 1 to 2. The third gene with a step size of 1 and the fourth gene with a step size of 1 represent the number of neurons in each LSTM layer. The number of neurons used ranged from 32 to 128. If the represented layer did not exist, the number of neurons was coded as 0. The fifth gene, with a step size of 1, and sixth gene with a step size of 1 represent the number of neurons in the entire junction layer. The number of neurons used ranged from 32 to 128. Similarly, if the represented layer did not exist, the number of neurons was coded as 0. The seventh gene with a step size of 0.1 represented the dropout rate of the dropout layer, and the number of dropout rates ranged from 0.1 to 0.5. The eighth gene, with a step size of 50, represented the number of epochs trained by the neural network. The number of epochs ranged from 50 to 250. In addition, there was only one neuron in the last layer, and the activation function was a sigmoid. To maintain the diversity of solutions, the dropout and epoch genes did not participate in cross-mutation. Fig 6 shows the chromosomes used in this study.

#### 3.2.2 Individual-ordering-based adaptive crossover method

An adaptive single-point crossing method was adopted in this study. First, an intersection was randomly set in a single chromosome. The two individuals then crossed the genes at the corresponding positions. The first and second numbers did not intersect, because they represent the number of layers. If the two numbers crossed, the number of subsequent neurons was affected. If the third to sixth positions exchanged genes with the number 0, they should not be crossed. The adaptive crossover probability is calculated based on Eq (5) mentioned in Section 2.3.

#### 3.2.3 Individual-ordering-based adaptive mutation method

The adaptive single-point mutation method was used in this study. This method adjusts only the individual values after the first two genes and randomly selects the values according to the corresponding range. The third to sixth values were randomly selected from 32 to 128. The seventh value was randomly selected from 0.1 to 0.5. The final values were randomly selected from 50 to 250. The adaptive mutation probability is calculated based on Eq (6) mentioned in Section 2.3.

#### 3.2.4 Elitist preservation strategy

The roulette method is used to select individuals based on the proportion of generated random numbers in the GA. However, the roulette method may mistakenly eliminate individuals with high fitness, and the fitness of each individual in this experiment was similar. The crossover and mutation operators used in the roulette method may destroy the high-average fitness pattern. Selection, crossover, and mutation may lead to erroneous elimination of the best individuals in the current population. In addition, this error may occur repeatedly during evolution. To avoid this phenomenon, we adopt an elite protection strategy, that is, copy the best individual (called an elite individual) in the population in the process of evolution into the next generation before crossover. Elites are individuals with the highest fitness value, best genetic structure, and good characteristics searched by a genetic algorithm in the process of population evolution. Therefore, the optimal individuals generated in the evolution process will not be lost or destroyed because of the selection, crossover, and mutation operations, which improves the ability of the algorithm to converge to global optimization.

#### 3.2.5 AGA training method

The first 82% of all stock data were used as training data and the last 18% as test data in the AGA-LSTM model. The specific steps of the adaptive genetic algorithm are as follows. First, the population was initialized, and the value of each chromosome was determined. Subsequently, the number of network layers, hidden neurons, and iterations of the LSTM neural network were determined. Next, the neural network was trained to predict the stock index. Finally, the prediction effect of the model is obtained by comparing the actual price of the sample with the predicted price of the model. In this study, the mean square error (MSE) of the model was used as the fitness function, and the smaller the MSE, the higher the prediction accuracy.

$$MSE=\frac{1}{n}\sum_{i=1}^{n}{\left(y_{i}-\hat{y}\right)}^{2},$$ (18)

where ${\hat{y}}_{i}$ is the predicted price of the model’s ith observation, y_*i*_ is the desired price, and n is the number of samples.

The roulette method was used to generate a next-generation population. The adaptive crossover and mutation operations are performed on the individuals of the new population to generate new individuals. This process is repeated to reach the maximum number of iterations, output the optimal individual, and retain the optimal LSTM model. Finally, the training data are processed in the optimal LSTM model to output the predicted price, which is then compared with the actual price.

### 3.3 Evaluation indicators

The evaluation indicators used in this study were the mean square error (MSE) [30–32], root mean square error (RMSE) [31–33], mean absolute error (MAE) [30–33], mean absolute percentage error (MAPE) [30–32], and goodness of fit (R^2^) of the model [33, 34]. RMSE is defined as

$$RMSE=\sqrt{\frac{1}{n}\sum_{i=1}^{n}{\left(y_{i}-{\hat{y}}_{i}\right)}^{2}}.$$ (19)

MAE is given as

$$MAE=\frac{1}{n}\sum_{i=1}^{n}\left|y_{i}-{\hat{y}}_{i}\right|.$$ (20)

MAPE is defined as

$$MAPE=\frac{1}{n}\sum_{i=1}^{n}\left|(y_{i}-{\hat{y}}_{i})/y_{i}\right|\times 100.$$ (21)

R^2^ is defined as

$$R^{2}=1-\frac{\sum_{i=1}^{n}{\left(y_{i}-{\hat{y}}_{i}\right)}^{2}}{\sum_{i=1}^{n}{\left(y_{i}-\bar{y}\right)}^{2}}.$$ (22)

The MSE, RMSE, MAE, and MAPE were used to measure the deviation between the actual and predicted values. The smaller the value, the closer the predicted value to the actual value. R^2^ was used to measure the degree of model fitting. The closer it was to 1, the better the model fitting.

## 4 Experimental results and comparison

### 4.1 Parameter setting

The hardware and software environment used in the experiment were as follows: the processor was an Intel (R) Core (TM) i5-9500 CPU with 8.0 GB memory; the system was Windows 10 (64 bit); the programming language version was Python 3.8.5; and the IDE was Jupyter 2021.8 in Visual Studio code extension. The AGA-LSTM, GA-LSTM, and LSTM were implemented in the Keras library with Tensorflow as the backend. Other machine learning (ML) models have been implemented in scikit-learn. This study used the first 82% of all stock data as training data and the last 18% as test data. Table 4 expresses the parameter set for the adaptive genetic algorithm.

**Table 4:** Parameters set for adaptive genetic algorithm.

Details of the parameters set	
Sequence length	50
Train_test_split	0.82
Batch_size	32
Loss	MSE
Optimizer	Adam
DNA_SIZE_MAX	8
POP_SIZE	20
N_GENERATION	50
P_c1_	0.9
P_c2_	0.6
P_m1_	0.1
P_m2_	0.5

### 4.2 Convergence comparison

#### 4.2.1 Convergence of AGA-LSTM algorithm

**Fig 7:**
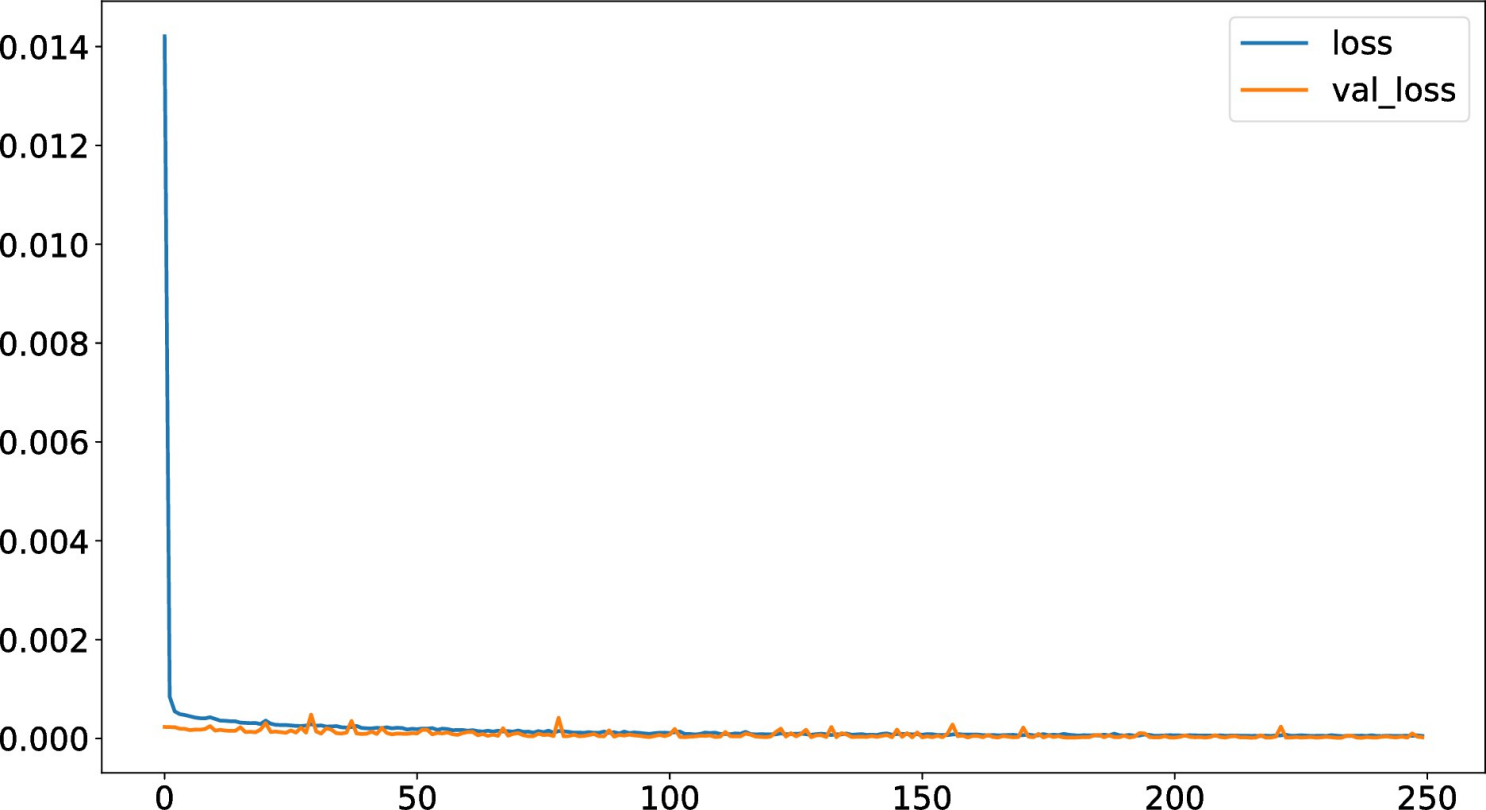
Error variation diagram of AGA algorithm.

The convergence of the algorithm is the key to improving its performance. The AGA-LSTM neural network was trained 250 times and its change pattern was observed. As shown in Fig 7, with an increase in training time, the error gradually converges, indicating that the algorithm is stable. This evolutionary method is suitable for the later stages of population evolution, as it can retain excellent individuals and achieve rapid convergence.

#### 4.2.2 Optimal individual

**Fig 8:**
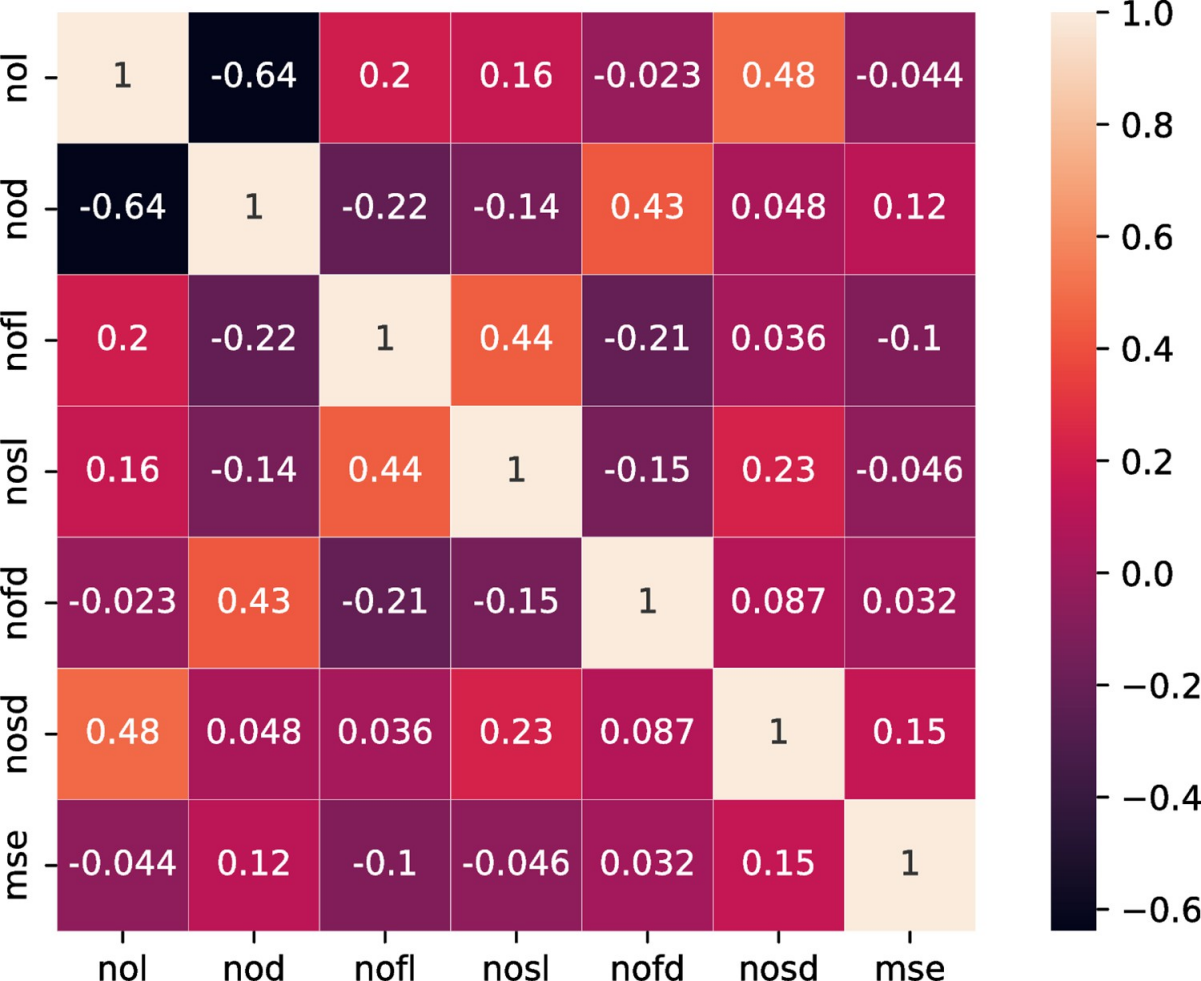
Correlation of 50 optimal combination parameters.

According to the statistics of 50 optimal individuals, as shown in Fig 8, the correlation coefficients of nod, nofl, nosd, and MSE are 0.12, -0.1 and 0.15, respectively, with weak correlation, and the correlation coefficients of nol, nofl, and nofd with MSE are -0.044, - 0.046 and 0.032, respectively, with little relevance.

**Fig 9:**
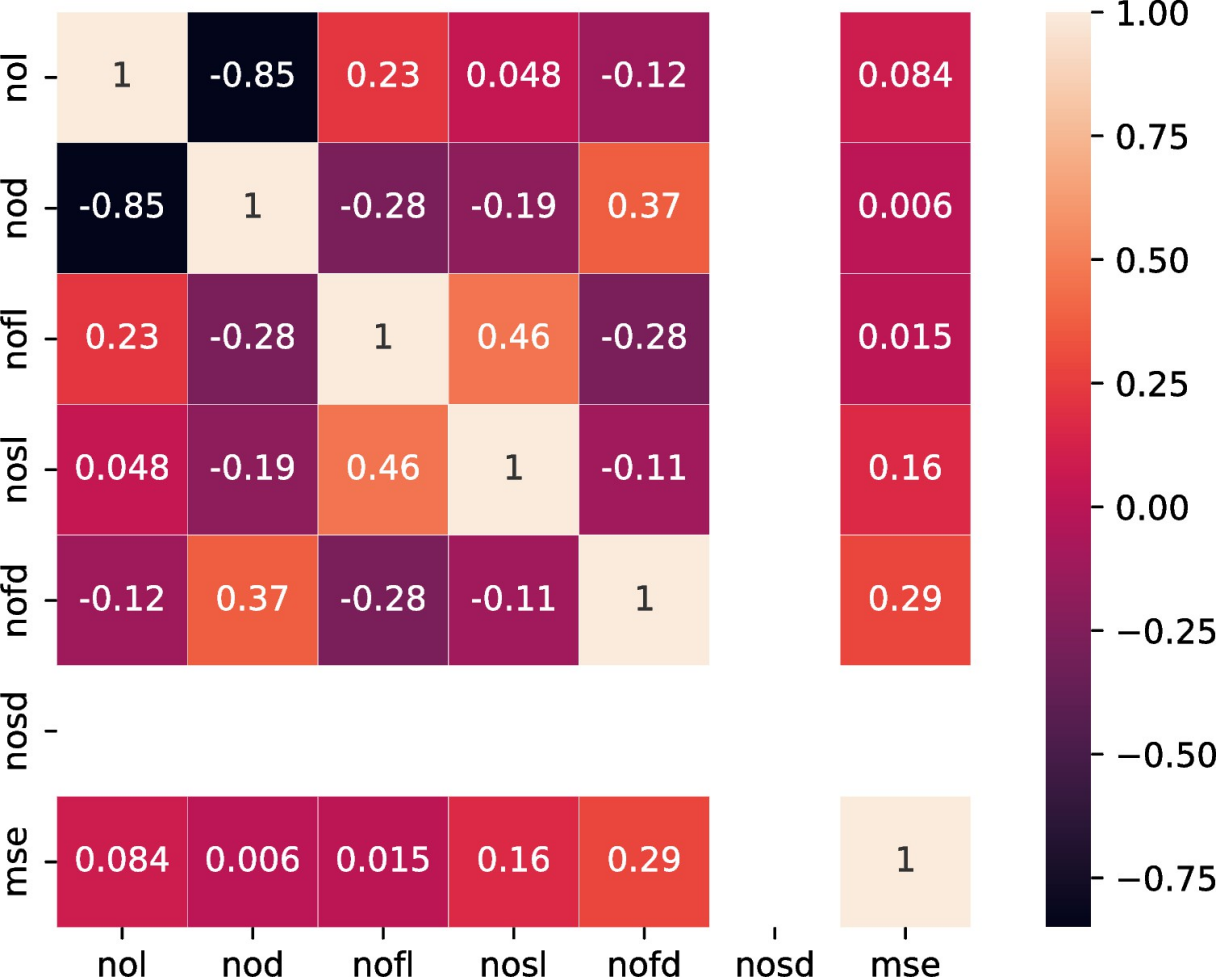
Correlation of 30 optimal combination parameters.

A fine comparison of the 30 optimal individual statistical parameters is shown in Fig 9; the correlation coefficients of nosl, nofd, and MSE are 0.16 and 0.29, respectively, with weak correlation; The correlation coefficients of nol, nod, nofl, and MSE were 0.084, 0.006 and 0.015, respectively, with little relevance.

A detailed comparison of the statistical parameters of the first 50 individuals and a statistical description of the prediction accuracy are shown in Table 5. Among the first 50 optimal value data generated by 50 iterations, the search coverage of nol and nod is 1 ~ 2; The search range of nofl, nosl, nofd, and nosd is 32 ~ 128; The search range of the six parameters is close to the full search space, and the error obtained is approximately the optimal solution. The minimum error was 5.23E-05 and the average error was 6.09E-05.

**Table 5:** Statistical description of 50 optimal parameter combinations.

nod	Nofl	nosl	nofd	Nosd	MSE
50	50	50	50	50	50
1.9	84.14	79.2	82.58	2.24	6.09E-05
0.30	23.57	28.16	30.50	15.84	4.45E-06
1	33	33	0	0	5.23E-05
2	71	53.5	58.75	0	5.77E-05
2	86	78.5	87.5	0	6.04E-05
2	104.5	100	105.5	0	6.56E-05
2	124	124	125	112	6.83E-05

A detailed comparison of the statistical parameters of the first 30 individuals and a statistical description of the prediction accuracy are shown in Table 6. In the first 30 optimal value data generated by 30 iterations, the search coverage of nol and nod was 1–2, the search range of nofl, nosl, nofd, and nosd was 32–128, the search range of the six parameters was close to the full search space, and the error obtained was approximately the optimal solution. The minimum error was 5.23E-05 and the average error was 5.77e-05.

**Table 6:** Statistical description of 30 optimal parameter combinations.

	Nol	nod	nofl	Nosl	nofd	nosd	MSE
**count**	30	30	30	30	30	30	30
**mean**	1.1	1.87	86.37	80.87	84.17	0	5.77E-5
**std**	0.31	0.36	25.5	26.8	31.31	0	2.67E-6
**min**	1	1	33	37	0	0	5.23E-5
**25%**	1	2	70.25	58.5	58	0	5.56E-5
**50%**	1	2	91	82	94	0	5.815E-5
**75%**	1	2	107	100	109.5	0	6.00E-5
**max**	2	2	124	122	124	0	6.17E-5

#### 4.2.3 Convergence comparison between AGA-LSTM and GA-LSTM

**Fig 10:**
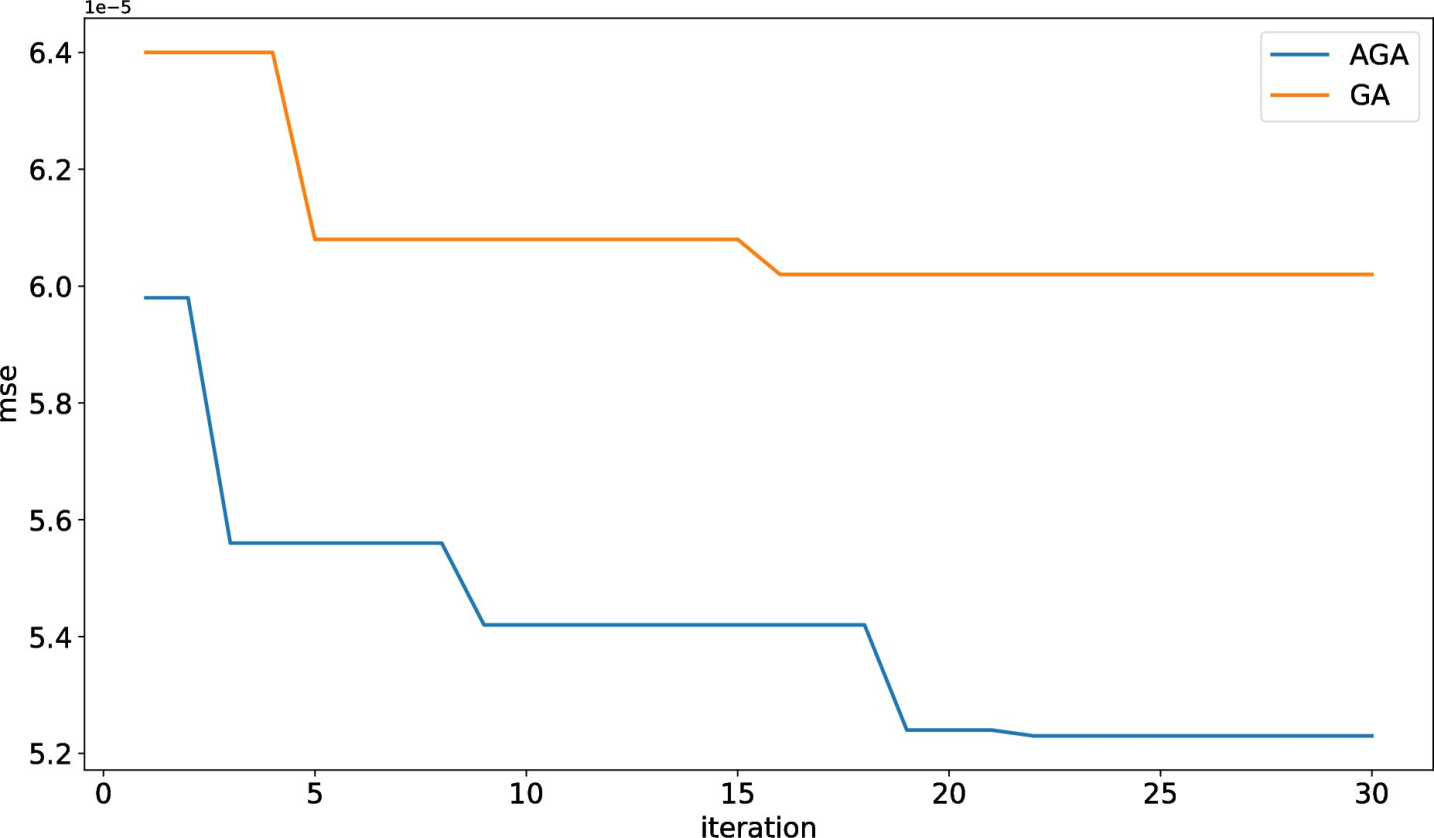
Fitness curve of AGA-LSTM and GA-LSTM.

As shown in Fig 10, AGA-LSTM, and GA-LSTM can quickly determine the approximate optimal solution in the search space. Compared with GA, the convergence effect of AGA shows little difference in the early and middle stages, but the convergence speed of AGA suddenly accelerates in the later stage. The results verify that the adaptive strategy proposed in this study improves the crossover and mutation rates of the algorithm in the later stages of evolution. This new optimization mode drives individuals to continuously evolve and helps the algorithm eliminate the old model and jump out of the local optimization.

**Fig 11:**
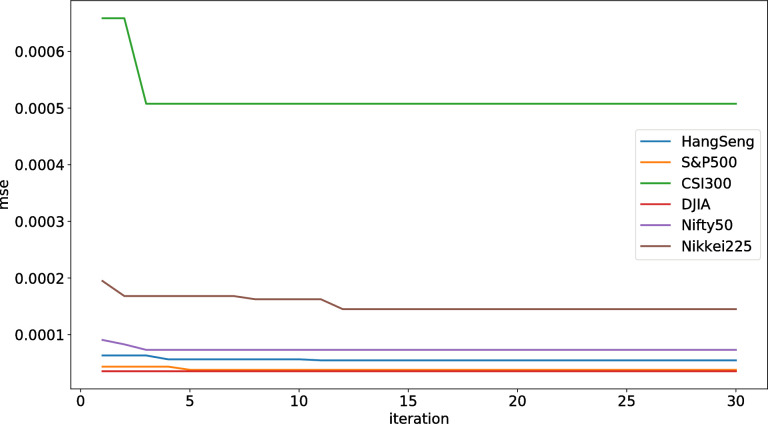
Convergence diagram of six stock indexes.

The convergence diagram of the six stock indices (Fig 11) shows that the AGA-LSTM model attained good convergence.

### 4.3 Prediction index and comparison

#### 4.3.1 Prediction chart

**Fig 12:**
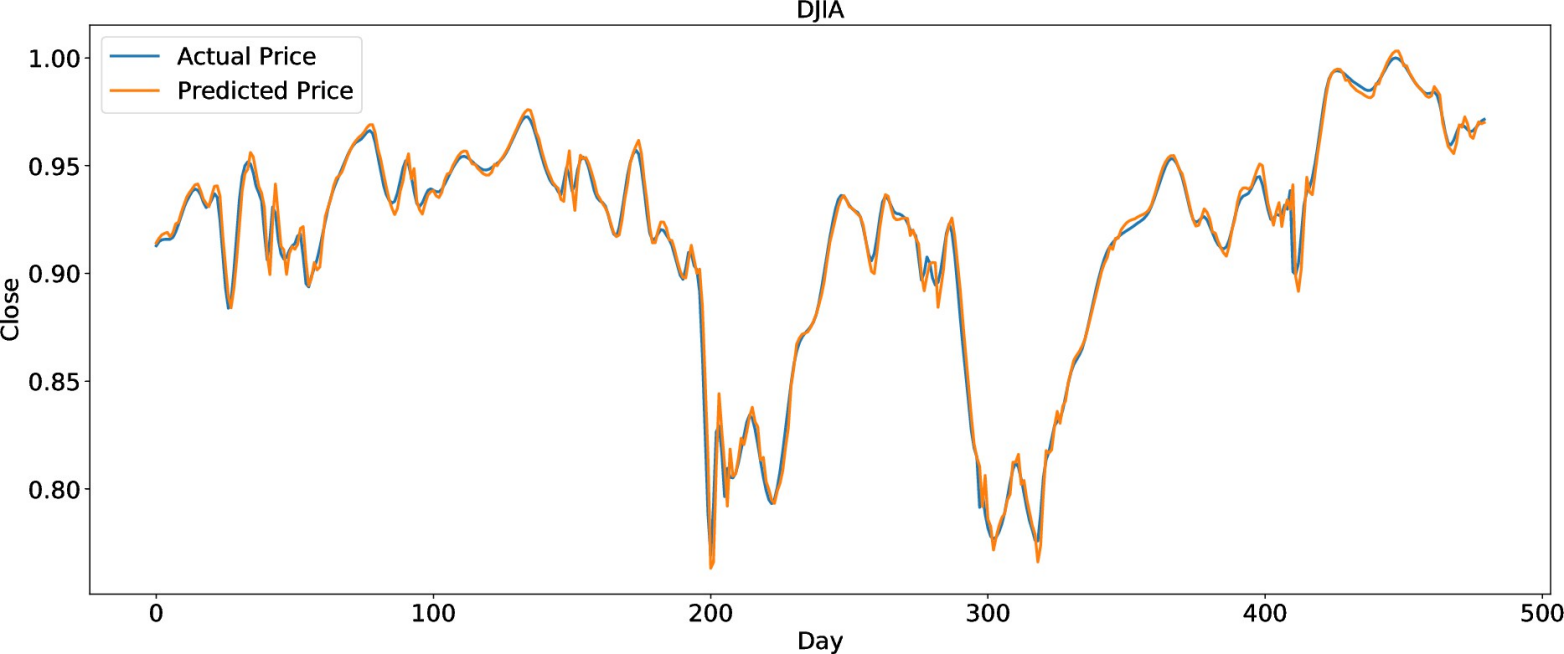
Prediction results of DJIA data set.

**Fig 13:**
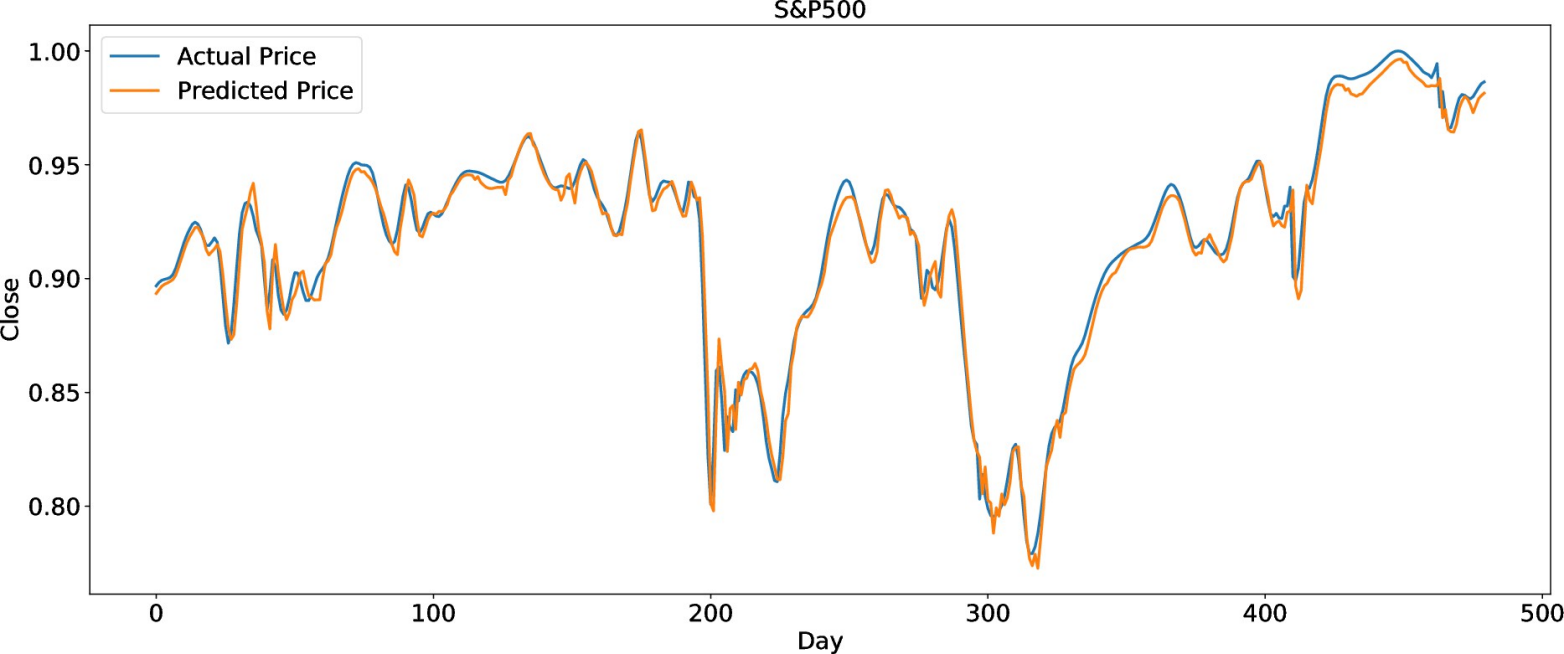
Prediction results of S&P data set.

**Fig 14:**
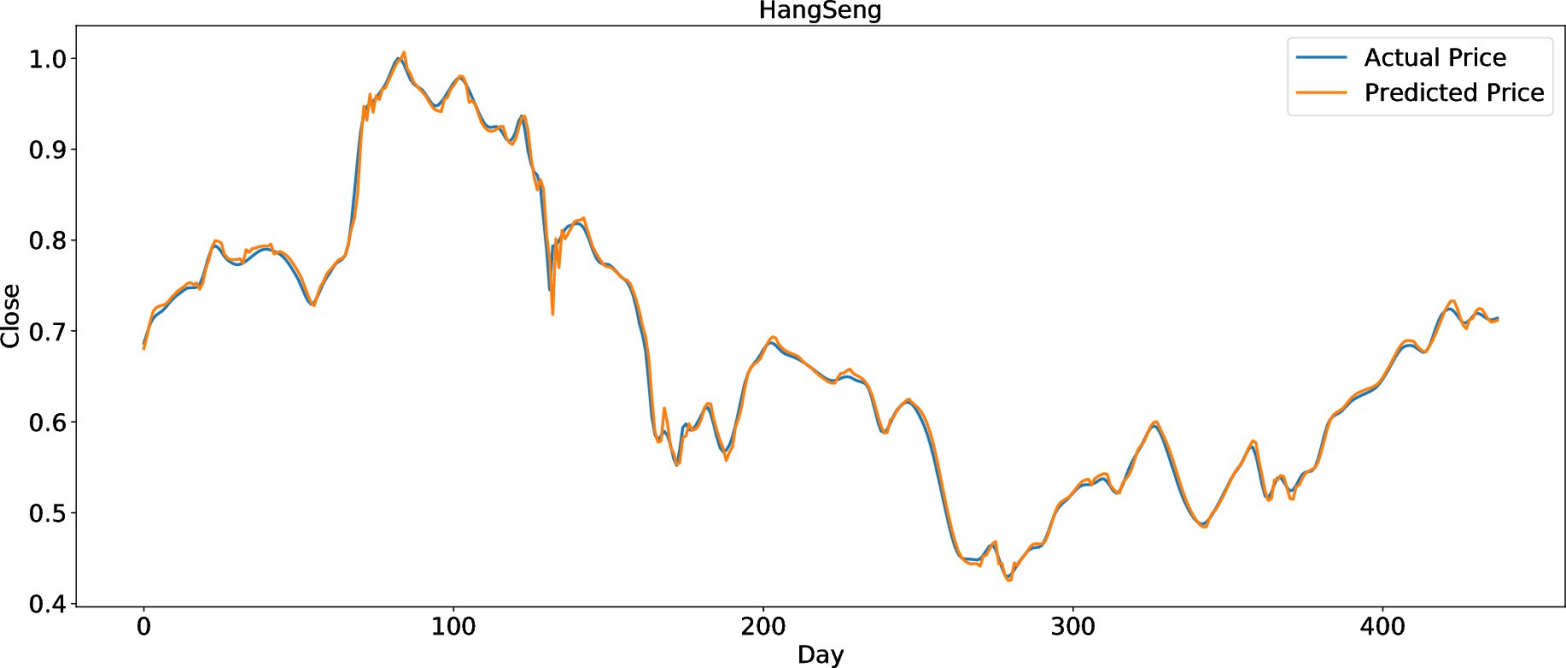
Prediction results of HangSeng data set.

**Fig 15:**
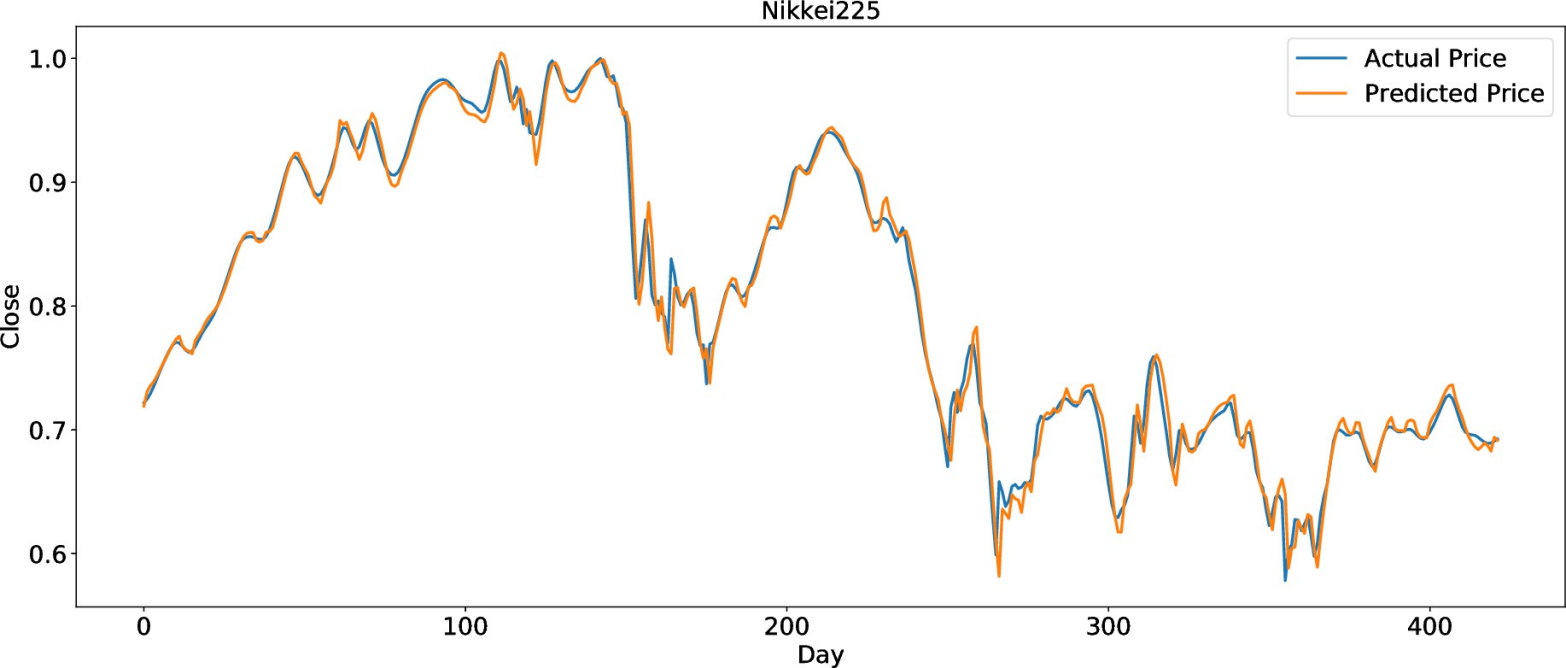
Prediction results of Nikkei225 data set.

**Fig 16:**
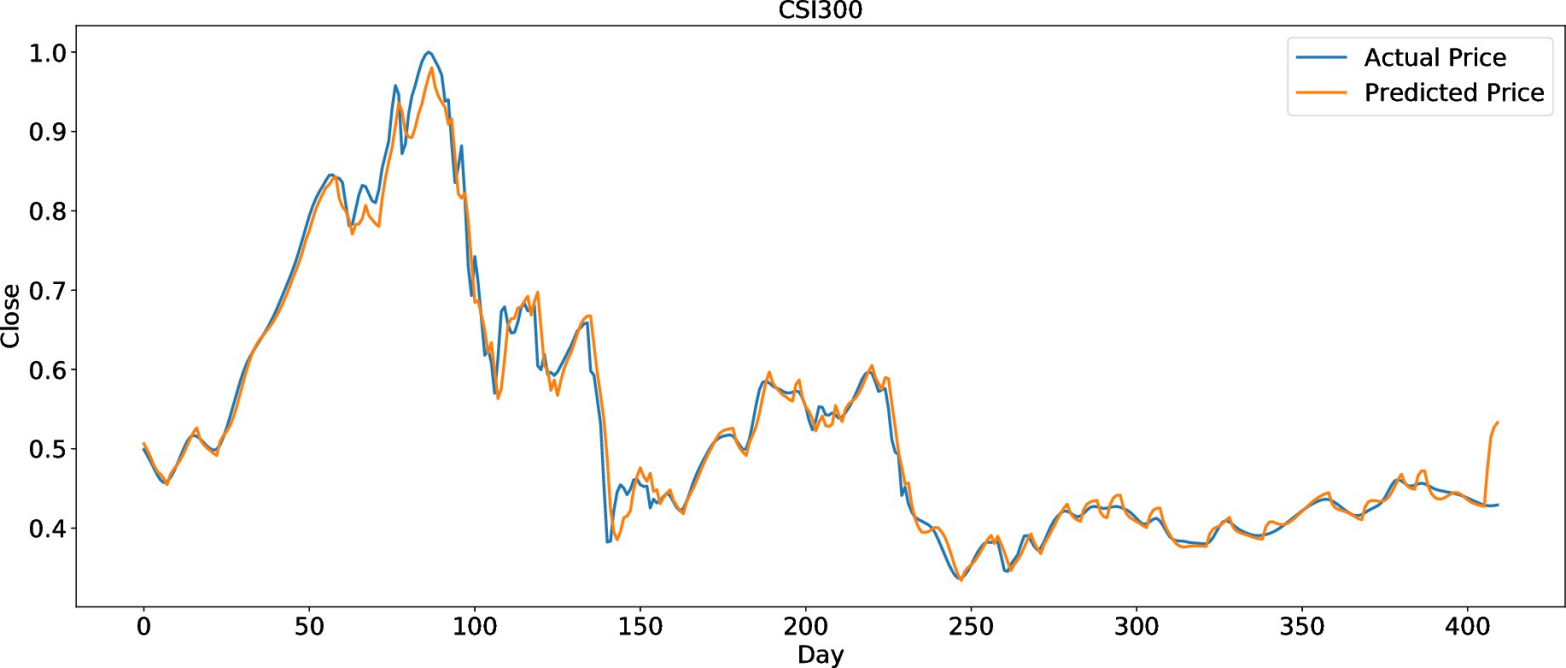
Prediction results of CSI300 data set.

**Fig 17:**
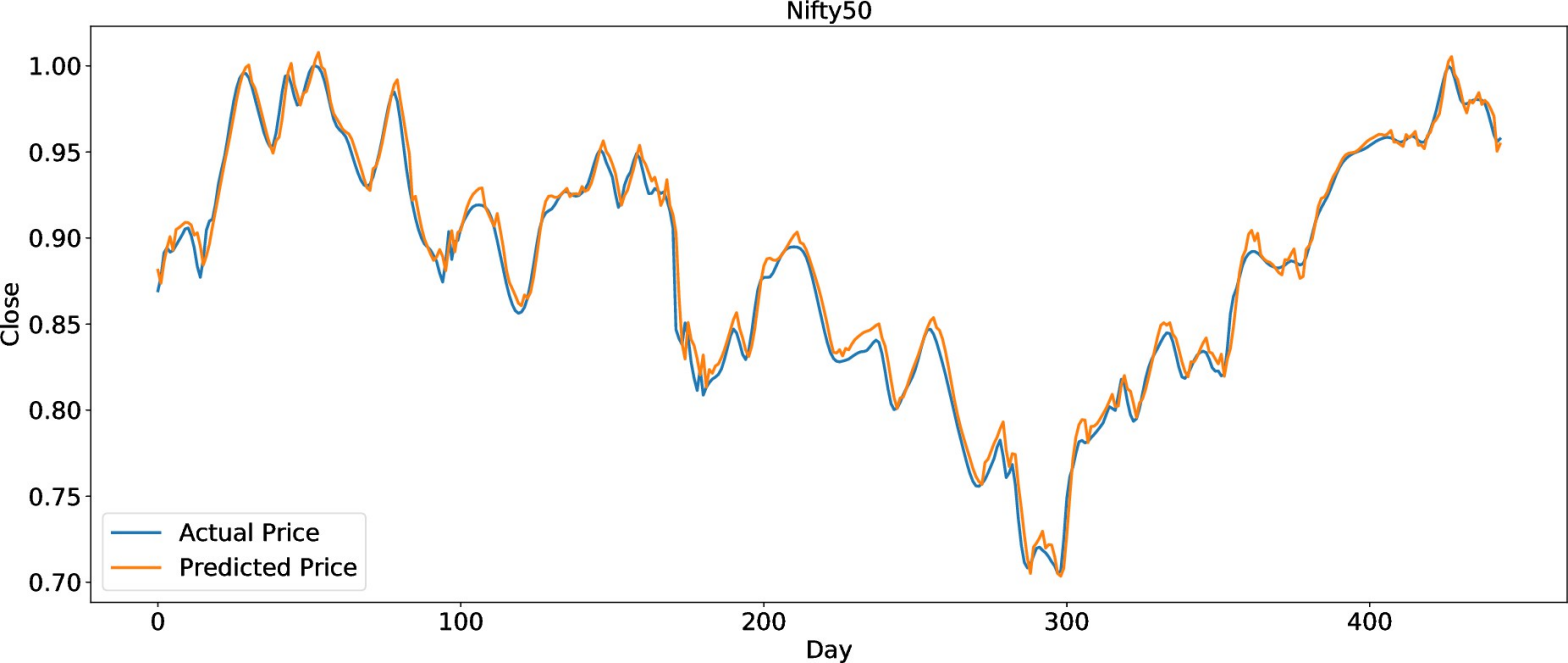
Prediction results of Nifty50 data set.

To clearly present the prediction performance of AGA-LSTM, this study uses the actual and predicted prices of the six stock indices. Figs 12–17 show the prediction results of the six stock indices attained by the AGA-LSTM model. As shown in the figures, the algorithm can fit the actual data well with a fast trend change, strong adaptability of the model, and high prediction accuracy.

#### 4.3.2 Comparison of the prediction effect with other deep learning models

**Fig 18:**
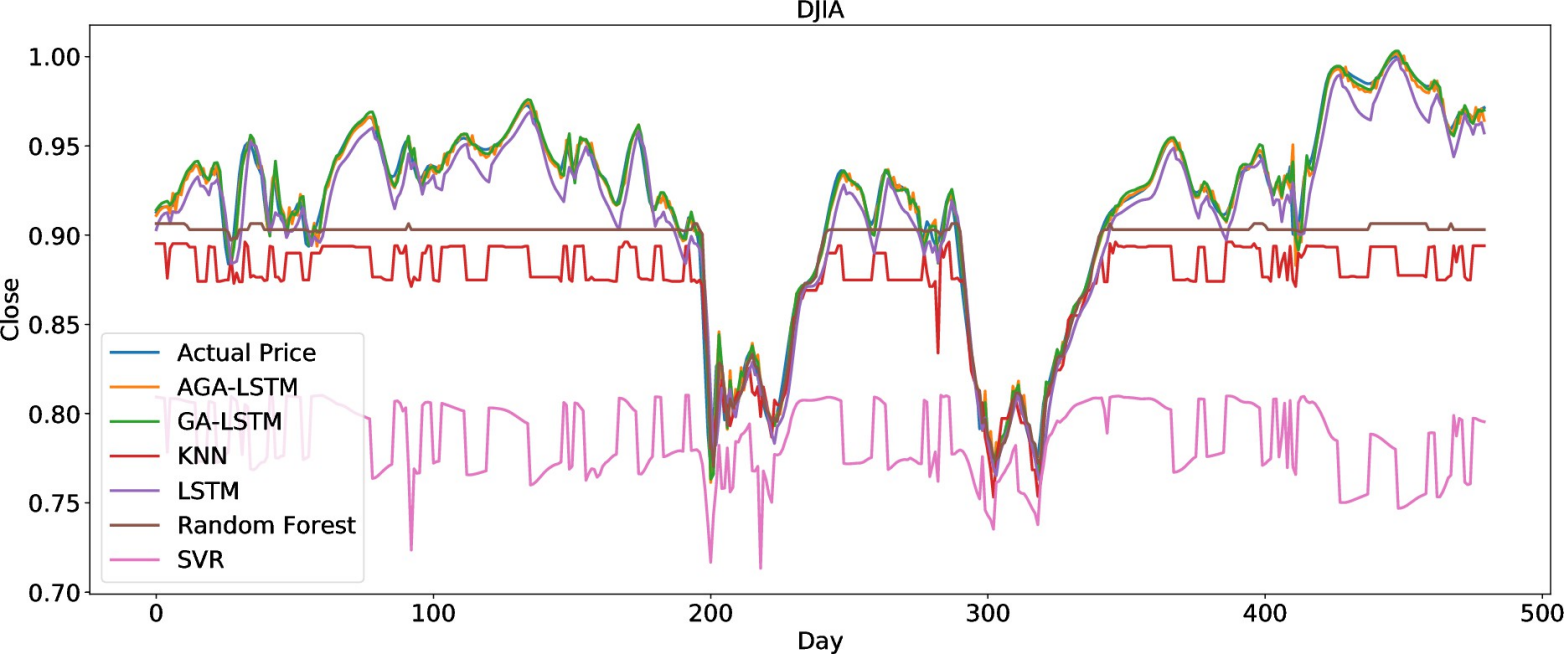
Comparison of AGA-LSTM and other DL models in DJIA data set.

**Fig 19:**
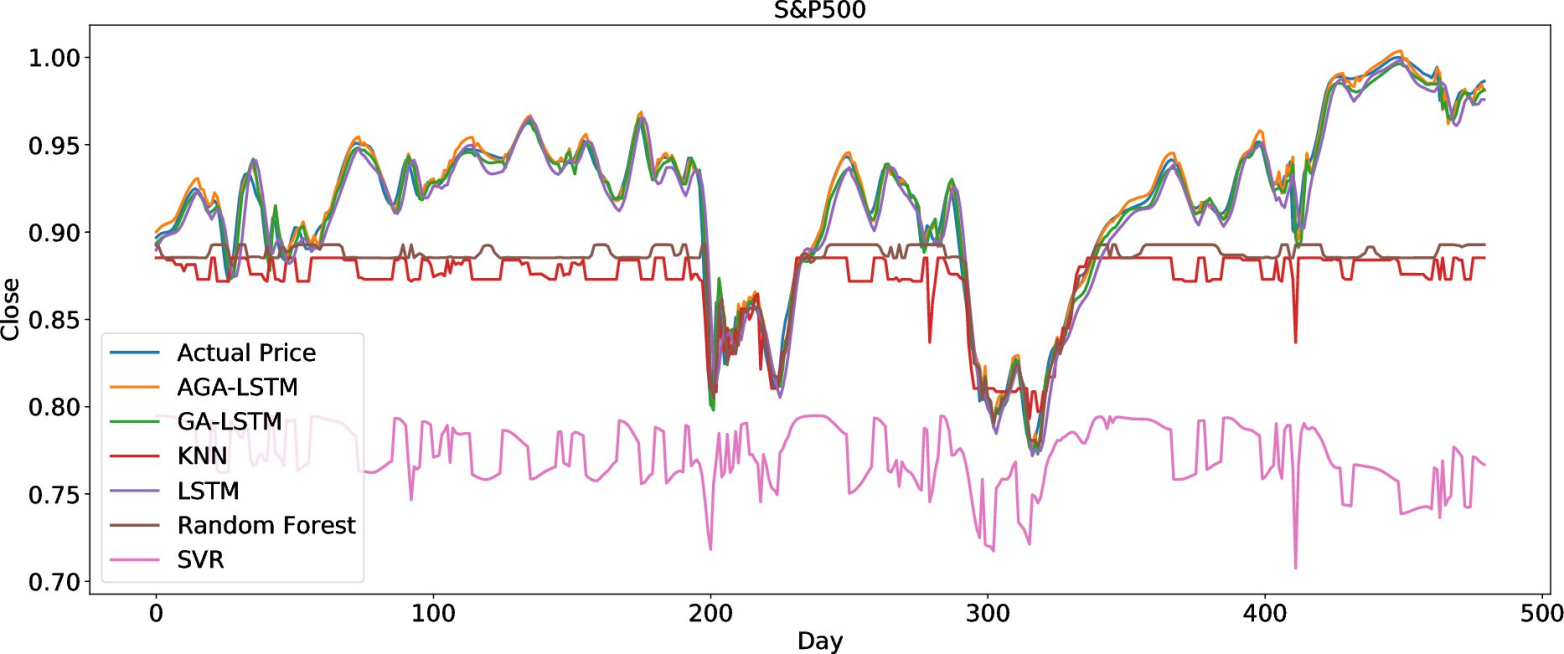
Comparison of AGA-LSTM and other DL models in S&P500 data set.

**Fig 20:**
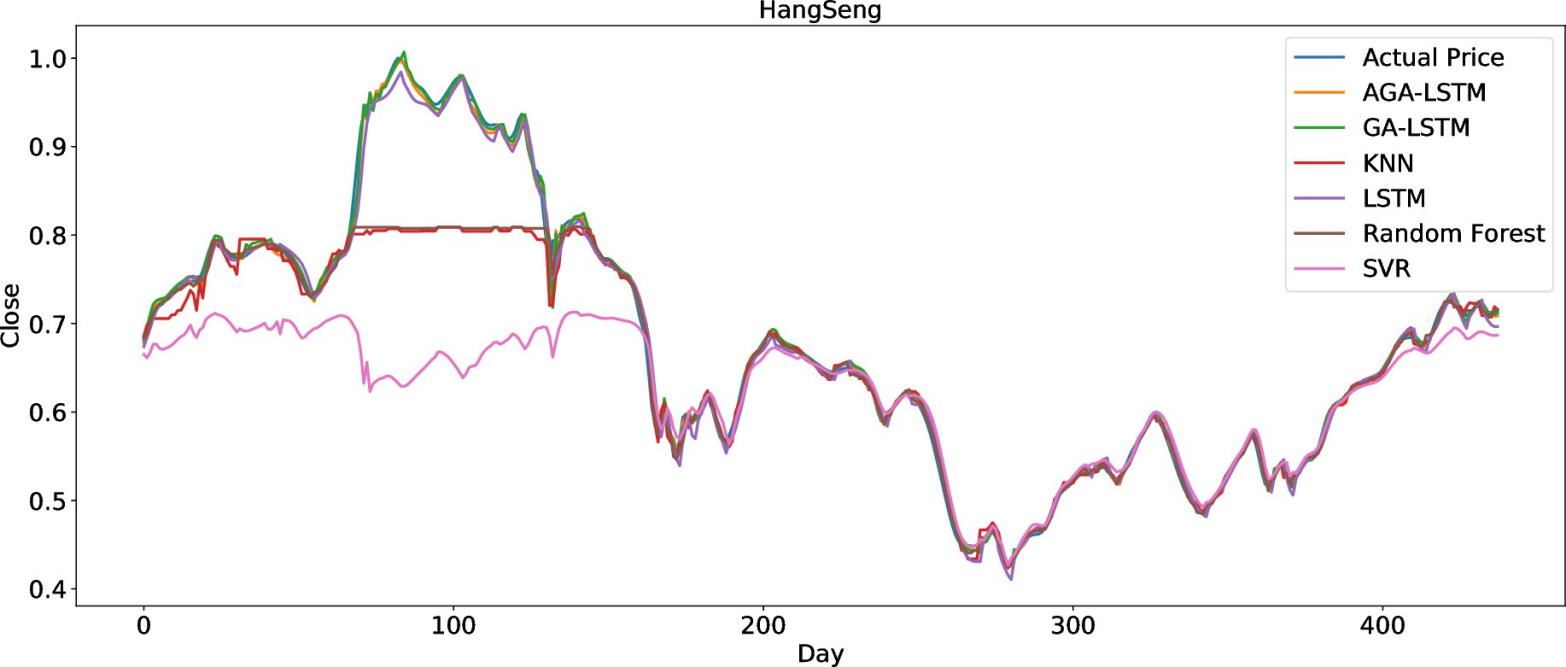
Comparison of AGA-LSTM and other DL models in HangSeng data set.

**Fig 21:**
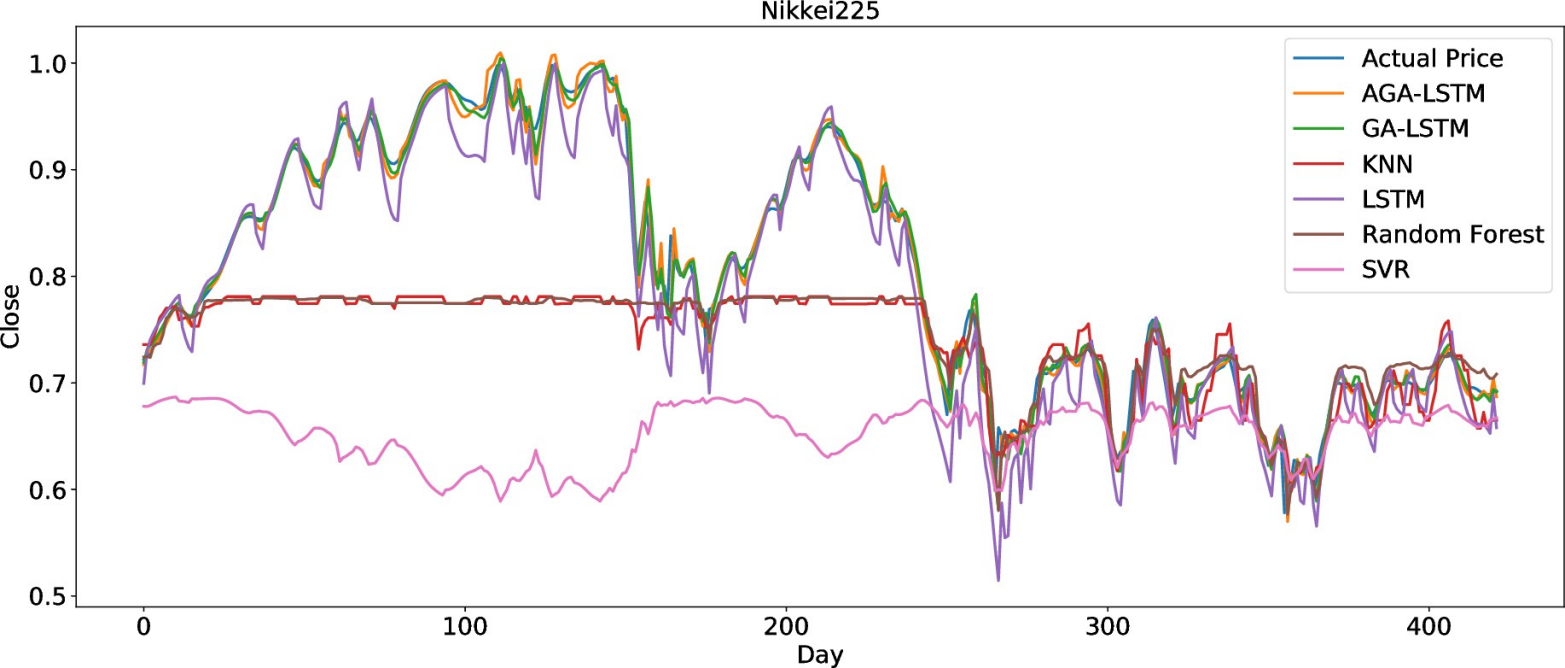
Comparison of AGA-LSTM and other DL models in Nikkei225 data set.

**Fig 22:**
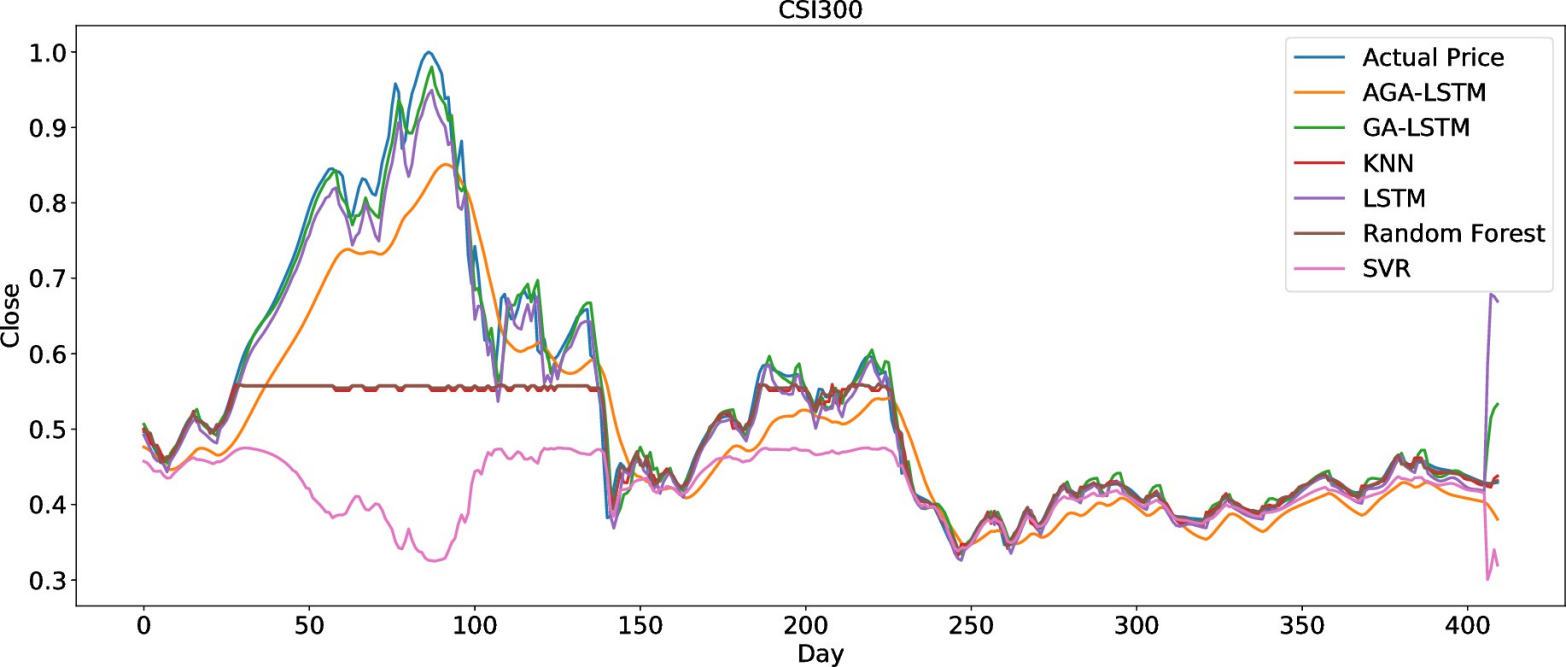
Comparison of AGA-LSTM and other DL models in CSI300 data set.

**Fig 23:**
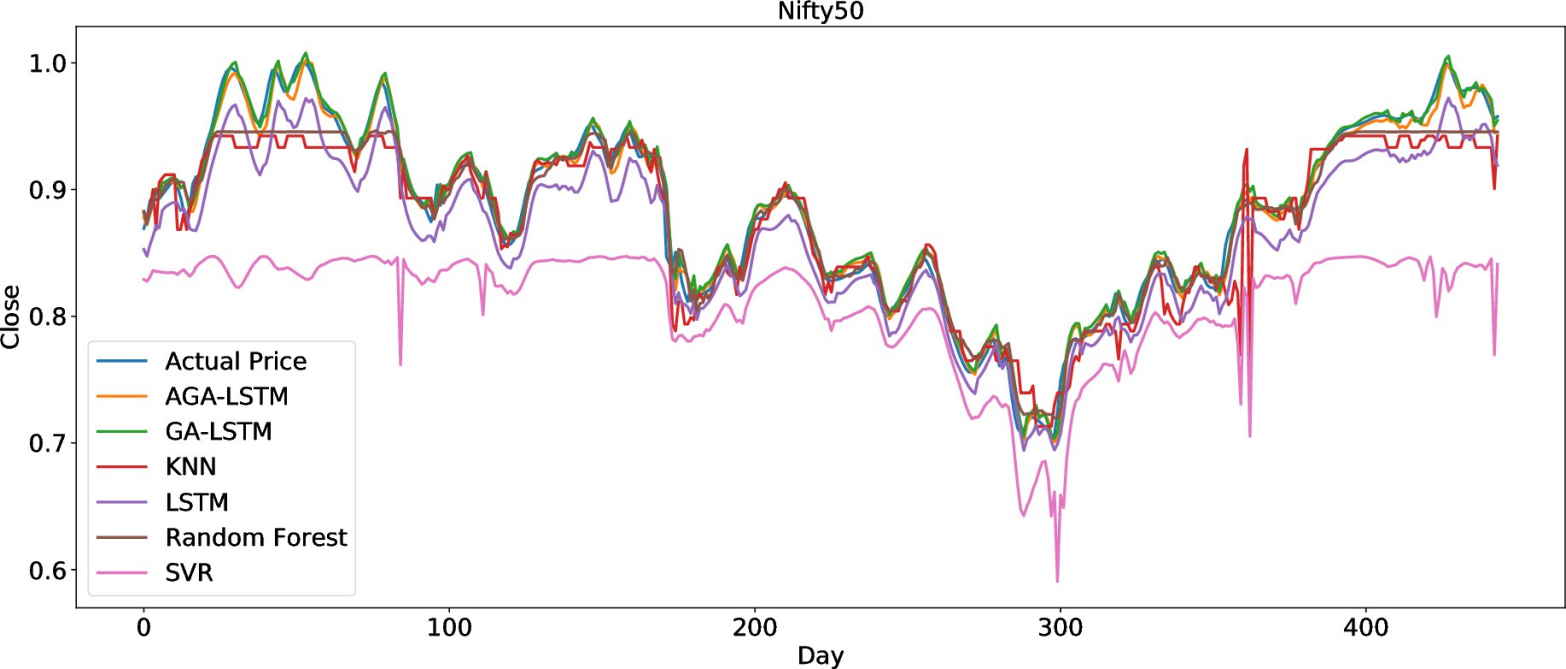
Comparison of AGA-LSTM and other DL models in Nifty50 data set.

The AGA-LSTM model used in this study was compared with GA-LSTM, SVM, KNN, random forest, LSTM, and other methods to predict the trend of the stock index. Figs 18–23 show the comparison of the AGA-LSTM and other DL models in six stock indexes. The figures illustrate the better prediction effect of AGA-LSTM compared with other deep learning models.

#### 4.3.3 Comparison of prediction indicators R^2^

The evaluation indices of the AGA-LSTM model, GA-LSTM, SVM, KNN, random forest, and LSTM for predicting stock trends are shown in Tables 7–12. In addition to the CSI300 dataset, AGA-LSTM has the smallest error index of MSE, RMSE, MAE, and MAPE and the largest error index of R^2^ on the other five stock index datasets. The AGA-LSTM model achieved the highest accuracy of 99.75% for the Hangseng stock index dataset. These indicators demonstrated the superiority of the proposed model. Specifically, the AGA-LSTM model is slightly better than the GA-LSTM model, but it is much better than the other deep learning models. The AGA-LSTM has good prediction performance and stability in the dataset of each stock index, which shows the good applicability and stability of the model.

**Table 7:** The evaluation indexes of AGA-LSTM model and other DL models in DJIA date set.

*DJIA*	MSE	RMSE	MAE	MAPE	R square
KNN	2.98E-03	5.46E-02	4.58E-02	4.850	-0.0654
RF	1.58E-03	3.98E-02	3.09E-02	3.260	0.438
SVR	2.01E-02	1.42E-01	1.32E-01	14.100	-6.201
LSTM	1.50E-04	1.22E-02	1.06E-02	6.292	0.946
AGA-LSTM	3.53E-05	5.94E-03	3.85E-03	6.245	0.987
GA-LSTM	3.07E-05	5.54E-03	3.57E-03	6.273	0.989

**Table 8:** The evaluation indexes of AGA-LSTM model and other DL models in S&P500 date set.

*S&P500*	MSE	RMSE	MAE	MAPE	R square
KNN	3.27E-03	5.72E-02	4.76E-02	5.049	-0.451
RF	2.58E-03	5.08E-02	4.06E-02	4.289	-0.148
SVR	2.35E-02	1.53E-01	1.45E-01	15.589	-9.407
LSTM	1.50E-04	1.22E-02	9.92E-03	5.740	0.937
AGA-LSTM	3.79E-05	6.15E-03	4.18E-03	5.683	0.983
GA-LSTM	4.26E-05	6.53E-03	4.78E-03	5.656	0.981

**Table 9:** The evaluation indexes of AGA-LSTM model and other DL models in HangSeng date set.

*HangSeng*	MSE	RMSE	MAE	MAPE	R square
KNN	6.53E-03	8.08E-02	6.29E-02	8.691	0.695
RF	2.95E-03	5.43E-02	2.33E-02	2.647	0.863
SVR	3.03E-02	1.74E-01	1.30E-01	17.061	-0.415
LSTM	7.24E-05	8.51E-03	5.81E-03	25.185	0.997
AGA-LSTM	5.23E-05	7.23E-03	4.52E-03	25.084	0.998
GA-LSTM	6.02E-05	7.76E-03	5.04E-03	25.281	0.997

**Table 10:** The evaluation indexes of AGA-LSTM model and other DL models in Nikkei225 date set.

*Nikkei225*	MSE	RMSE	MAE	MAPE	R square
KNN	1.03E-02	1.02E-01	7.24E-02	8.057	0.224
RF	1.02E-02	1.01E-01	7.07E-02	7.831	0.234
SVR	3.98E-02	1.99E-01	1.52E-01	17.028	-1.988
LSTM	4.70E-04	2.17E-02	1.49E-02	16.847	0.965
AGA-LSTM	1.45E-04	1.20E-02	7.85E-03	16.772	0.989
GA-LSTM	1.48E-04	1.22E-02	7.87E-03	16.714	0.988

**Table 11:** The evaluation indexes of AGA-LSTM model and other DL models in CSI300 date set.

*CSI300*	MSE	RMSE	MAE	MAPE	R square
KNN	1.46E-02	1.21E-01	5.71E-02	7.459	0.416
RF	1.45E-02	1.20E-01	5.64E-02	7.303	0.419
SVR	3.83E-02	1.96E-01	1.09E-01	15.363	-0.536
LSTM	1.97E-03	4.44E-02	1.85E-02	30.041	0.921
AGA-LSTM	5.08E-04	2.25E-02	1.47E-02	30.603	0.980
GA-LSTM	4.69E-04	2.17E-02	1.32E-02	30.507	0.981

**Table 12:** The evaluation indexes of AGA-LSTM model and other DL models in Nifty50 date set.

*Nifty50*	MSE	RMSE	MAE	MAPE	R square
KNN	5.60E-04	2.37E-02	1.61E-02	1.778	0.890
RF	2.81E-04	1.68E-02	1.12E-02	1.226	0.945
SVR	7.04E-03	8.39E-02	7.36E-02	8.093	-0.383
LSTM	5.59E-04	2.36E-02	2.20E-02	9.113	0.890
AGA-LSTM	6.53E-05	8.08E-03	6.18E-03	9.289	0.987
GA-LSTM	7.31E-05	8.55E-03	6.45E-03	9.213	0.986

## 5 Conclusion

The main contribution of this study is the optimization of the LSTM model using an adaptive genetic algorithm. The optimization mechanism based on individual raking can automatically adjust the network structure of the model and slightly tune the combination of hyperparameters, which significantly reduces the frequencies of the hyperparameter adjustment. This study constructs a high-precision model of AGA-LSTM stock price prediction. We performed a quantitative analysis of the six stock indices. The results show that the proposed model performs better than the LSTM alone. The predictive performance of AGA-LSTM is also better than that of GA-LSTM and other machine learning models. The methods used in the model are as follows. Real number coding was performed to find the optimal solution directly in the solution space. The adaptive crossover and mutation probability were adjusted to accelerate the speed of finding the optimal solution and hyperparameter combination. These optimization methods made the model achieve the predominant prediction objectives. In this study, because the fitness difference of each individual was not obvious, an adaptive algorithm based on individual fitness ranking was proposed to improve the speed of finding the optimal solution. In addition, the convergence efficiency of AGA was higher than that of GA. To obtain better accuracy, there are many directions to explore in the future. For example, using more features of big data, such as the emotional characteristics of investors in the stock market, may improve prediction accuracy. With the further application of deep learning, high-dimensional data mining and time-series prediction, considering the influence of multi-state correlation will also become the next research direction.

## Supporting information

S1 Data (RAR)
